# Ceramic-in-Polymer Hybrid Electrolytes with Enhanced Electrochemical Performance

**DOI:** 10.1021/acsami.2c13408

**Published:** 2022-11-21

**Authors:** Gerrit Michael Overhoff, Md Yusuf Ali, Jan-Paul Brinkmann, Peter Lennartz, Hans Orthner, Mohaned Hammad, Hartmut Wiggers, Martin Winter, Gunther Brunklaus

**Affiliations:** † Helmholtz Institute Münster, IEK-12, Forschungszentrum Jülich GmbH, Corrensstreet 46, 48149Münster, Germany; ‡ Institute for Combustion and Gas Dynamics—Reactive Fluids, University of Duisburg-Essen, Carl-Benz-Straße 199, 47057Duisburg, Germany; § Institute for Combustion and Gas Dynamics—Particle Science and Technology, University of Duisburg-Essen, Carl-Benz-Straße 199, 47057Duisburg, Germany; ∥ CENIDE, Center for Nanointegration, University of Duisburg-Essen, Carl-Benz-Straße 199, 47057Duisburg, Germany; ⊥ MEET Battery Research Center, Institute of Physical Chemistry, University of Münster, Corrensstreet 46, 48149Münster, Germany

**Keywords:** lithium metal batteries, composite electrolytes, polymer electrolytes, single-ion conductor, ceramic-in-polymer, functionalized LATP

## Abstract

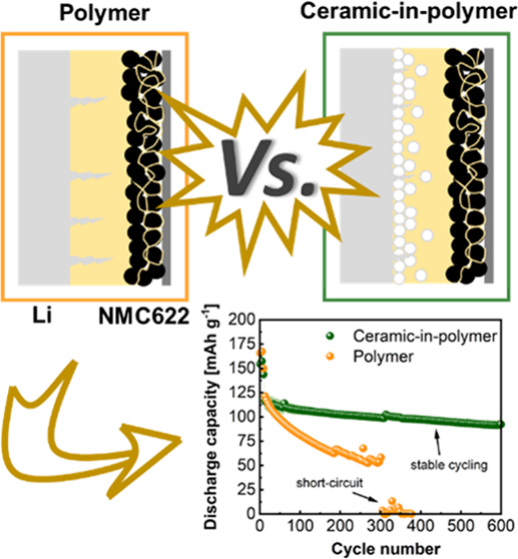

Polymer electrolytes are attractive candidates to boost the application of rechargeable lithium metal batteries. Single-ion conducting polymers may reduce polarization and lithium dendrite growth, though these materials could be mechanically overly rigid, thus requiring ion mobilizers such as organic solvents to foster transport of Li ions. An inhomogeneous mobilizer distribution and occurrence of preferential Li transport pathways eventually yield favored spots for Li plating, thereby imposing additional mechanical stress and even premature cell short circuits. In this work, we explored ceramic-in-polymer hybrid electrolytes consisting of polymer blends of single-ion conducting polymer and PVdF-HFP, including EC/PC as swelling agents and silane-functionalized LATP particles. The hybrid electrolyte features an oxide-rich layer that notably stabilizes the interphase toward Li metal, enabling single-side lithium deposition for over 700 h at a current density of 0.1 mA cm^–2^. The incorporated oxide particles significantly reduce the natural solvent uptake from 140 to 38 wt % despite maintaining reasonably high ionic conductivities. Its electrochemical performance was evaluated in LiNi_0.6_Co_0.2_Mn_0.2_O_2_ (NMC622)||Li metal cells, exhibiting impressive capacity retention over 300 cycles. Notably, very thin LiNbO_3_ coating of the cathode material further boosts the cycling stability, resulting in an overall capacity retention of 78% over more than 600 cycles, clearly highlighting the potential of hybrid electrolyte concepts.

## 1 Introduction

Due to increasing demands for batteries in emerging fields such as electromobility, materials with high energy and power densities are required. Lithium (Li) metal anodes combined with high-voltage and high-capacity cathode materials such as LiNi_0.*x*_Co_0.*y*_Mn_0.*z*_O_2_ (NMCxyz) could meet these requirements,^1,2^ despite that the growth of high-surface area lithium (HSAL) deposits or “dendrites” upon cycling may impose safety risks.^1,3,4^ Solid electrolytes in principle could withstand or suppress penetration by lithium “dendrites” provided that they afford sufficient mechanical stability (moduli > 1 MPa). Indeed, polymers comprise a very promising solid electrolyte materials class and are highly flexible, thus, in principle, providing good contacts to active electrode materials. Also, they are readily processed, rendering them available in many varieties including bioderived materials.^5−7^ To date, the most commonly exploited solid polymer electrolyte, poly(ethylene oxide)/LiTFSI, suffers from modest room-temperature ionic conductivity, which can be attributed to its high degree of crystallinity and low Li^+^ transference number (*t*_Li^*+*^_ ≤ 0.3).^8^ To boost the unfavorably low *t*_Li^*+*^_, single-ion conducting polymers can be applied, where anions are attached to either the polymer backbone or sidechains offering *t*_Li^*+*^_ values of up to ≈1 similarly to inorganic electrolytes.^9^ Theoretically, this should avoid Li “dendrite” formation, considering both eq 1
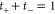
1and the notion that the proposed “dendrite” initiation time (Sand’s time) τ is reversely proportional to the anion transference number *t_–_*.^10,11^ If all of the anions are attached to rigid aromatic polymer backbones, blending with mechanically more flexible linear polymers such as PVdF-HFP is eventually required to achieve free-standing polymer electrolyte membranes. Additionally, single-ion conducting polymers are typically plasticized with salt-free organic solvents or ionic liquids or mixtures thereof^12^ that act as “molecular transporter” of Li ions, in this way cushioning the impact of rather limited polymer chain mobilities. A significant advantage of “*quasi*-solid” or gel-type polymer electrolytes constitutes their superior ionic conductivity (>1 mS cm^–1^ at r.t.), though this often requires an uptake of >100 wt %, which might pose safety risks.^13−17^ Previously, we reported on single-ion conducting polysulfonamine (PSA)–PVdF-HFP blend electrolytes soaked with up to 130 wt % of an ethylene carbonate/propylene carbonate (EC/PC) mixture.^13,18^ The blend polymer exhibited advantageous characteristics, including cyclability against high-voltage NMC-type cathode materials. Nevertheless, in view of current efforts and roadmaps to develop all solid-state batteries, reducing the solvent uptake of these polymer systems remains preferable. A promising way to improve *t*_Li^*+*^_ and reduce the solvent content is based on the ceramic-in-polymer (CIP) approach, particularly when utilizing active lithium-ion conducting fillers such as NaSICON-type Li_1.2_Al_0.2_Ti_1.8_(PO_4_)_3_ (LATP)^19^ or garnet-type Li_7_La_3_Zr_2_O_12_ (LLZO), respectively.^20^ Upon implementation of such composite polymer electrolytes (CPEs), new Li^+^ transport pathways are theoretically accessible either due to Li^+^ transport through the bulk ceramic or at the interface between polymer and ceramic domains.^21−23^ Note that especially the latter is considered to afford highly conductive Li^+^ transport.^24,25^ However, preferential Li^+^ pathways may be affected by interactions with the polymer matrix, ceramic composition and content as well as the presence of additional plasticizer/mobilizer agents.^26^ A synergistic effect combining plasticizers such as tetraglyme or PC with ceramics within a CPE recently demonstrated a boosted Li^+^ conductivity, though sufficient long-term cycling in full cells was missing, rendering further works necessary.^27,28^ In addition to a homogenous distribution of ceramic particles within the polymer matrix, CIP materials can be designed as layered or gradient structures.^29−32^ A ceramic-rich layer at the Li metal electrode may regulate Li^+^ flux during deposition, even at a capacity utilization of 15 mAh cm^–2^,^30^ or form a layer with ultrahigh mechanical strength to mitigate Li dendrite growth.^31^ Zhu et al. showed that a BaTiO_3_-rich layer electrospun at the cathode generates a protective film and stabilizes the interphase.^32^ However, the application of several coatings may require additional processing steps and the introduction of additional interfaces can increase the overall cell resistance.

Herein, we report an approach to fabricate layered hybrid electrolytes consisting of PSA, PVdF-HFP, and LATP particles. The latter are obtained *via* spray-flame synthesis (SFS), which is an effective one-step procedure to prepare multicomponent materials. SFS enables a scalable and robust production of high-purity nanoparticles with controlled chemical/phase composition, crystallinity, shape, and size.^33,34^ The materials synthesized by SFS are widely applied in fields, such as catalysis,^35^ quantum dot synthesis,^36^ sensors,^37^ cosmetics,^38^ and batteries.^39^ Note that LLZO synthesis based on SFS was reported earlier;^40,41^ here, we employed a similar SFS reactor to prepare LATP nanoparticles. To enable joint processing of particles and polymers, as well as to promote strong electrostatic bonding between particles and the partially negatively charged polymer backbone, the nanoparticles were functionalized with 3-aminopropyltriethoxysilane (APTES) by exploiting silane surface modification, yielding a positive zeta (ζ) potential for the particles by introducing NH_2_/–NH_3_^+^ groups. Membranes of the hybrid electrolyte are obtained from solvent casting of the ceramic particles together with polymer components in *N*-methyl-2-pyrrolidone (NMP) as the solvent with a high boiling point. During the evaporation of the solvent, APTES-LATP particles partly accumulate at the bottom of the membrane, in this way yielding oxide-rich layers. These *in situ* generated layers enable Li metal single-sided deposition for more than 700 h without any cell short circuit. Furthermore, the maximal solvent uptake of the hybrid polymer blend is reduced from 130 to only 38 wt % while maintaining a reasonably high ionic conductivity of 0.7 mS cm^–1^ at 40 °C. Moreover, the electrochemical cycling performance of NMC622||Li cells is evaluated, revealing highly promising capacity retention over 600 cycles at 0.5C, whereas the corresponding blend polymers without the presence of functionalized LATP particles suffer from severe losses of specific capacity.

## 2 Experimental Section

### 2.1 Materials

*p*-Toluenesulfonyl chloride (97%), *p*-toluenesulfonyl amide (97%), lithium hydroxide monohydrate, potassium permanganate, calcium chloride, *N*-methyl-2-pyrrolidone (NMP, anhydrous, 99.8%), pyridine (anhydrous, 99.8%), lithium aluminum hydride (95%), tetrahydrofuran (THF, anhydrous, 99.8%), lithium bis(trimethylsilyl) amide solution (1 M in THF), tributyl phosphate (≥99%), and titanium(IV) isopropoxide (97%) were purchased from Sigma-Aldrich. Aluminum nitrate (Al(NO_3_)_3_ × 9H_2_O), 3-(aminopropyl)triethoxysilane, 1-propanol, and propionic acid were acquired from Merck. 4,4-(Hexa-fluoroisopropylidene)dianiline (98%) was obtained from TCI Europe, poly(vinylidene difluoride-co-hexafluoropropylene) (PVdF-HFP, Kynar FLEX LBG) from *Arkema*, and lithium nitrate (LiNO_3_ × *x*H_2_O) from Alfa Aesar. Concentrated hydrochloric acid, methanol, dimethyl sulfoxide (DMSO), and triphenyl phosphite were acquired from VWR. Ethylene carbonate, propylene carbonate, and NMC622 were purchased from BASF. Carbon black (Super C65) was obtained from Imerys Graphite & Carbon and polyvinylidene difluoride (PVdF, Solef 5130) from Solvay. Prior to use, calcium chloride was dried at 180 °C under reduced pressure (10^–3^ mbar) for 48 h.

### 2.2 Sample Preparation

#### 2.2.1 Synthesis of PSA

Polymer PSA was produced following a previously reported protocol.^13^ Details of the synthesis route of PSA (Figure S1) are included in the supporting information as well as respective ^1^H-NMR spectra (Figures S2–S6). The synthesized PSA has a molecular weight distribution *M*_n_ of 64 kg mol^–1^ and a *M*_w_ of 98 kg mol^–1^ (Figure S7).

#### 2.2.2 Synthesis of APTES-LATP Particles

Lithium nitrate, aluminum nitrate, titanium(IV) isopropoxide, and tributyl phosphate were used as the SFS precursor for Li, Al, Ti, and phosphate, respectively. Li and Al precursors were added to a mixture (50:50 v/v) of 1-propanol/propionic acid and further heated at 70 °C until a clear solution was obtained. Then, appropriate amounts of Ti and phosphate precursor were added into the solution. The overall solution concentration was kept at 0.3 M, and the solution was pumped into a custom-made spray-flame reactor. The reactor setup was discussed in detail in a previous publication.^8^ Briefly, a sinter-metal-stabilized pilot flame (CH_4_/O_2_ 1:8 v/v) surrounding a central two-fluid spray nozzle (10 slm O_2_ for atomization, precursor solution feeding rate of 2 mL min^–1^) was used to ignite the spray whereupon the subsequent precursor evaporation and decomposition results in nanoparticle nucleation and growth downstream the spray flame. Coaxial sheath gas (140 slm) was used to stabilize the flame and additional quench gas (240 slm) injected downstream of the reaction zone was used to cool down the reactor and carry the product to the filter. The as-synthesized samples were mildly calcined in a horizontal tube furnace at 700 °C for 1 h under an O_2_ atmosphere to remove residual unburnt hydrocarbons and to form the intended NaSICON-type LATP phase. The surface functionalization of the calcined powder was carried out by dispersing the powder in distilled water, and the pH of the dispersion was adjusted between 3.5 and 4 using 0.1 M HCl solution. APTES was added (APTES/sample ratio 1:1 w/w) to the above dispersion solution. The solution was heated at 70 °C under constant reflux overnight for 14 h, centrifuged (10 000 rpm, 15 min), washed three times, and finally dried at 130 °C under reduced pressure (10^–6^ mbar) for 72 h.

#### 2.2.3 Membrane Fabrication

PVdF-HFP was dissolved in 4 mL of NMP, and PSA was slowly added to the solution under stirring (a ratio of PVdF-HFP/PSA was 1:3). Then, APTES-LATP (5, 10, 20, 33 wt %) was given to the polymer mixture, and the resulting suspension was stirred for 2 h and sonicated (UP100H, Hielscher) for 1 h. The suspension was subsequently cast into a PTFE dish and dried at 80 °C for 24 h. To remove residual solvent inside the membranes, they were further dried under reduced pressure (10^–3^ mbar) at 80 °C for 24 h. Finally, the membranes were swollen with an EC/PC mixture (1:1 v/v), resulting in overall membrane thicknesses of 90–110 μm. Membranes with LATP and Al_2_O_3_ were prepared similarly; in the case of membranes containing only polymer, the sonication step was skipped.

#### 2.2.4 Electrode Preparation

NMC622 electrodes were prepared by wet casting of electrode paste containing 90 wt % NMC622 (as-obtained or coated with 0.5 wt % LiNbO_3_), 7 wt % conductive agent (carbon black, Super C65), and 3 wt % PVdF. After dissolving PVdF in NMP, the other components were added and mixed using a Thinky planetary mixer at 1500 rpm for 10 min. Afterward, the electrode paste was cast onto aluminum foil (20 μm) using a doctor blade (Zeiss, Swiss) set to a wet-film thickness of 50 μm. The electrode sheets were dried at 80 °C, calendered to a porosity of ≈30%, and then punched out to circular disks (⌀12 mm). The average mass loading of the electrodes was 2.1 mg cm^–2^ at a film thickness of 14 μm. To further improve contacts between the polymer membrane and cathode active material, the electrodes were spin-coated using 30 μL of PSA solution (10 wt % PSA in NMP). Therefore, the solution was added dropwise onto electrode disks while increasing the rotation speed stepwise to 120 rps and holding it for 120 s. All of the electrodes were dried to remove residual NMP.

### 2.3 Material Characterization

Thermogravimetric analysis (TGA) was carried out with a Netsch STA 449 F1 (NETZSCH-Gerätebau GmbH, Germany) under air with a heating rate of 10 K min^–1^ up to 1200 °C. The samples were pressed into 5 mm width pellets for the measurements.

The ζ potential of the catalysts was determined by a Malvern Zetasizer (Malvern Panalytical, United Kingdom). The crystal structures of LATP, APTES-LATP, PSAb, and the composite electrolyte were studied using a Bruker D8 Advance device equipped with Cu Kα X-ray tubes in the 2θ range of 10–60°.

Scanning electron microscopy (SEM) investigations were performed in a Carl Zeiss AURIGA CrossBeam workstation with a Schottky field emission gun and an energy-dispersive X-ray (EDX) unit by Oxford Instruments. Images were obtained with a secondary electron detector at an acceleration voltage of 3 kV and a working distance of 3 mm. EDX was done at an accelerating voltage of 20 kV and a working distance of 3 mm. Prior to the measurement, all samples were coated with a very thin gold layer to reduce static surface charging of the respective materials.

The morphology and particle size of LATP were determined using a transmission electron microscope (TEM, JEM-2200FS, JEOL) by first dispersing the particles in isopropanol and drop casting on copper TEM grids. Then, the dried TEM grid was mounted on a sample holder for further characterization by TEM. The mean particle size of the TEM obtained images was measured by ImageJ software.

X-ray photoelectron spectroscopy (XPS) measurements were performed on a VersaProbe II (Ulvac-Phi) using a monochromatic Al X-ray source (1486.6 eV) operated at 15 kV and 13.2 W. The emission angle between the sample and analyzer was kept constant at 45°. CASA-XPS software was used to fit the data, and the C 1s signal at 284.8 eV was used as an internal reference data point to calibrate other elemental spectra. Sputtering of the samples was done for 240, 480, 720, and 960 s with an Ar ion beam at a voltage of 3.2 V and an etching depth of ∼3 nm for each sputtering step.

Mechanical properties of the membranes were measured with an MCR-102 rheometer (Anton Paar Inc.) in oscillatory mode with parallel plates (diameter of 15 mm). A frequency sweep was carried out at a constant strain amplitude of 0.05% (a ratio of deflection path to gap height) between 400 and 0.1 rad s^–1^.

### 2.4 Electrochemical Investigations

Ionic conductivities were measured by electrochemical impedance spectroscopy (EIS). Polymer membranes (⌀8 or 10 mm) of known thickness were placed between two polished stainless-steel electrodes in coin cells (CR2032). Prior to the measurements, the cells were heated to 70 °C to improve interfacial contact between the electrodes and electrolyte. All of the cells were measured on an Autolab PGStat302N potentiostat with a frequency analyzer FRA32 (Deutsche Metrohm GmbH & Co., KG, Germany) at temperatures ranging from 0 to 70 °C (10 °C steps) and in a frequency range of 1 MHz to 1 Hz, applying a voltage amplitude of 10 mV. The limiting current density, *t*_Li^*+*^_, and Li plating experiments were conducted on a VMP3 multichannel potentiostat (Bio-Logic Science Instruments). The membranes were sandwiched between two Li metal electrodes (Albemarle, roll-pressed from 500 to 350 μm) in a coin-cell setup with a symmetric (two) electrode configuration.^42^ To determine the limiting current density, the voltage was increased with 0.02 mV s^–1^ at 40 °C. For calculation of *t*_Li^*+*^_, the method proposed by Evans et al. was exploited.^43^ Before and after applying a polarization voltage Δ*V* of 10 mV, the impedance of the cell was measured and *t*_Li^*+*^_ was derived according to the formula
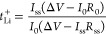
2where *I*_0_ and *I*_ss_ denote the initial and steady-state currents, while *R*_0_ and *R*_ss_ are the initial and steady-state resistances of the interface. Oxidative/reductive stability was measured in a three-electrode configuration (Swagelok)^42^ using copper or platinum as working electrodes and lithium metal as both counter and reference electrodes at a scan rate of 0.1 mV s^–1^. Constant current (CC) cycling was performed on an LBT20084 battery cycler (Arbin Instruments) with an integrated Autolab PGSTAT204 in the voltage range of 3.0–4.3 V at 40 °C. The cycling starts with a formation step consisting of cell charge/discharge at rates of 2 × 0.05C, 2 × 0.1C, and 2 × 0.05C, respectively, followed by a stepwise increase until 0.5C (assuming a theoretical specific capacity of 180 mAh g^–1^ for NMC622). EIS data were recorded after the first formation cycle, at the end of the formation, as well as after 30, 60, 100, and 300 cycles.

### 2.5 Distribution of Relaxation Times

The impedance data were fitted based on distribution of relaxation times (DRT) and equivalent circuit modeling with the software RelaxIS by rhd-instruments. An appropriate equivalent circuit model was built based on the DRT analysis, which resolves and fits the measured spectrum in the frequency (or time) domain. DRT analysis was performed with a regularization parameter of λ = 10^–5^, which is a compromise between oversmoothening (too large values) and overfitting (too small values). The amount of authentic peaks (here with at least 30% peak intensity relative to the actual maximum intensity) was taken as a rough estimation for the amount of R-CPE elements to be implemented in a meaningful equivalent circuit model. Peaks below the threshold or with insufficient resolution were combined and taken as one (broadly distributed) R-CPE element. In the case of NMC||Li full cells, the high-frequency R-CPE element was substituted with an R-C element, as the fitting resulted in an exponent of the complex CPE function of α = 1 (ideal R-C element).

## 3 Results and Discussion

### 3.1 Particle Description and Membrane Fabrication

**Figure 1:**
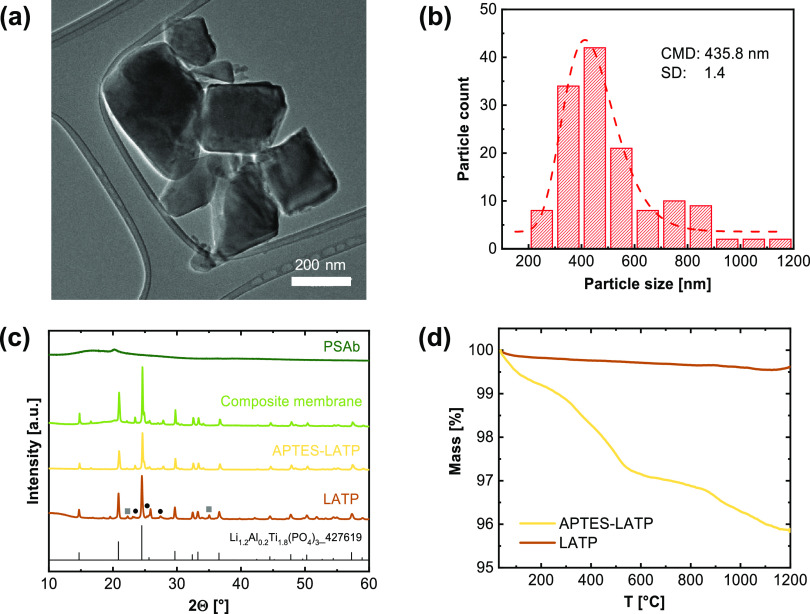
(a) TEM image of APTES-LATP, (b) particle size distribution after calcination, (c) XRD patterns of LATP powder, APTES-LATP, polymer mixture PSAb, and the hybrid membrane with 20 wt % APTES-LATP (■ AlPO_4_, ● TiP_2_O_7_ phase), and (d) TGA profiles before and after modification with APTES.

To establish insights into the particle size distribution of the as-synthesized LATP particles, TEM measurements were employed. After production by SFS, the particles show the expected lognormal particle size distribution with a count median diameter (CMD) of about 8 nm and a geometric standard deviation σ_g_ of 0.7 (see lognormal fit to the histogram of particle sizes in Figure S8). However, upon the following calcination step, the nanoparticles aggregate to larger structures as demonstrated in the TEM image of the particles (Figure 1a), which also results in an increase of the CMD to 435.8 nm and a geometric standard deviation σ_g_ of 1.3 (Figure 1b). After annealing the particles at 800 °C in an O_2_ atmosphere for 1 h, the desired LATP phase was formed almost quantitatively (bottom graph, Figure 1c) though minor reflexes indicate the presence of AlPO_4_ (**■**) and TiP_2_O_7_ (**●**) phases. The particles produced in this way have a negative ζ potential, which made it difficult to process them with also negatively charged backbone of the PSA polymer due to electrostatic repulsion. Therefore, a positive surface potential was set by surface functionalization of the annealed particles using APTES. TGA measurements were performed to monitor the successful modification of the LATP particle surfaces with APTES (Figure 1d). While the sample with annealed LATP particles does not lose any significant mass over the temperature range of 30–1200 °C, a decrease of 4 wt % is noted for APTES-LATP particles. ζ potential measurements of annealed and APTES-functionalized particles were done in aqueous media, revealing a change from −52 to +31 mV upon modification. During the modification process, the crystalline structure of the LATP particles remained intact, as demonstrated by the corresponding XRD patterns shown in Figure 1c. After each step (APTES functionalization, blending with the polymer), the characteristic signals of the anticipated LATP crystal structure of Li_1.2_Al_0.2_Ti_1.8_(PO_4_)_3_ are clearly detected. In the case of the polymer blend, a broad bulge in the XRD spectra indicates highly amorphous polymer domains, where merely a small reflex at 2θ = 20° is attributed to the presence of crystalline PVdF-HFP phases.^44^

**Figure 2:**
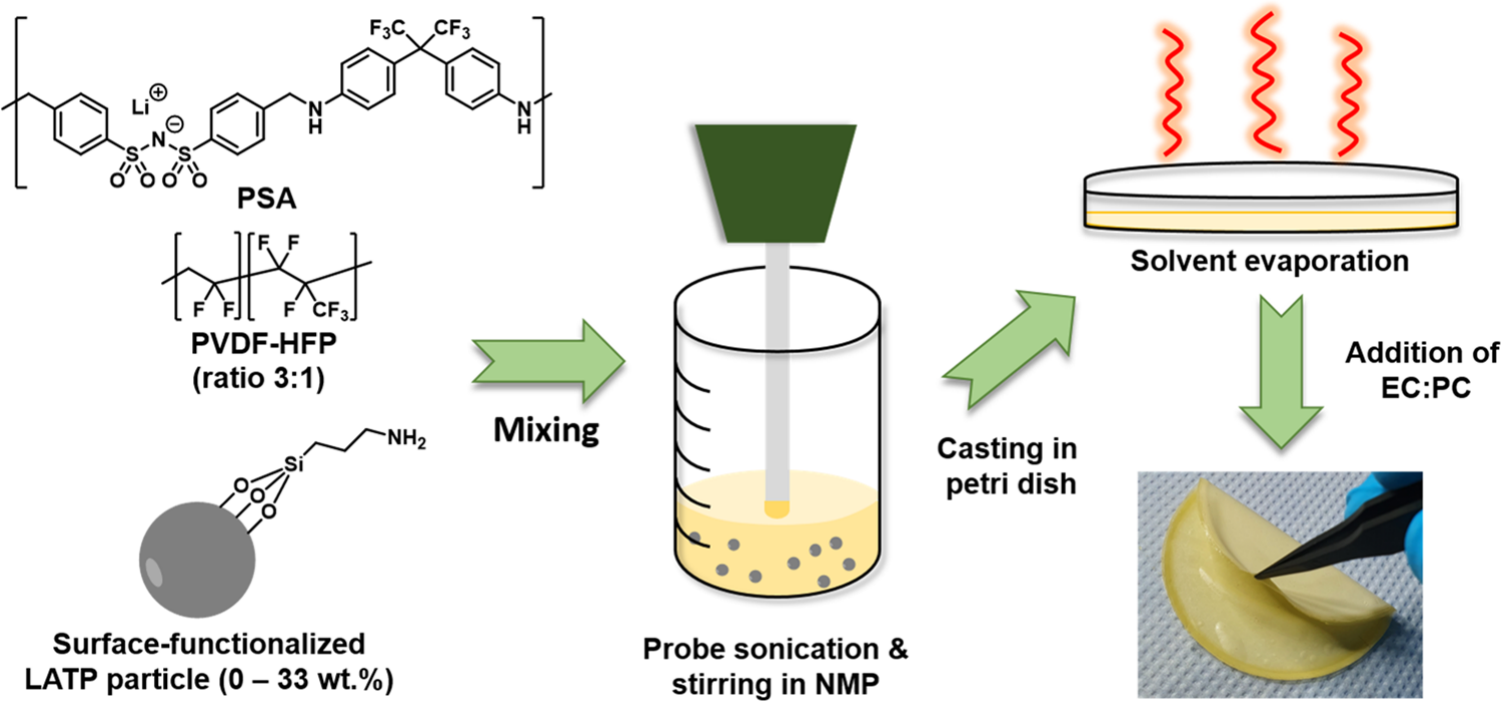
Schematic view of the membrane fabrication process and a snapshot of the resulting flexible membrane.

The detailed process of hybrid membrane manufacture is schematically shown in Figure 2. PSA denotes a single-ion conducting polymer, and PVdF-HFP is added to enhance the mechanical stability of the resulting blend membranes. Suitable blend compositions of PSA and PVdF-HFP required to achieve high ionic conductivity and mechanical stability were studied previously.^18^ For reasonably high ionic conductivities, an EC/PC mixture (1:1 v/v) is added to the membrane.

### 3.2 Properties of the Polymer/Oxide Membranes

**Figure 3:**
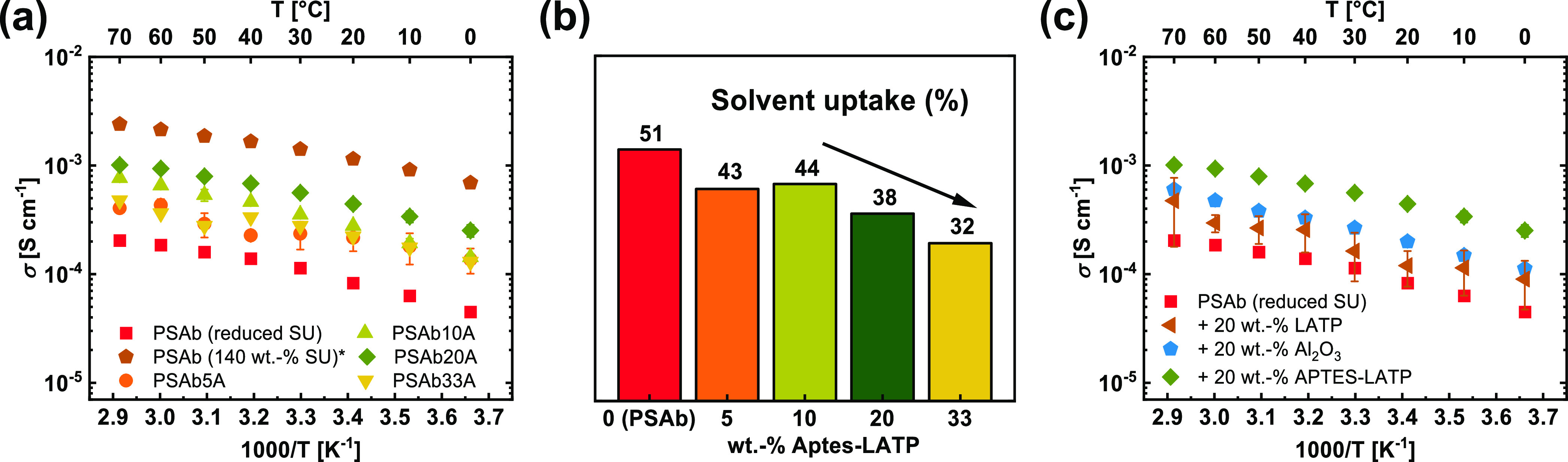
(a) Temperature-dependent ionic conductivity of hybrid electrolytes with different compositions of PSAb and APTES-LATP particles and (b) solvent uptake of the respective membranes. (c) Comparison of ionic conductivity of hybrid electrolytes with different oxide materials. *Data from Borzutzki et al.^13^

Optimal compositions of the polymer/oxide hybrid electrolytes were derived from varying the respective ratio of PSAb and APTES-LATP, considering concentrations listed in Table 1. Notably, ionic conductivity plays a crucial role for solid electrolytes since a high ionic conductivity is indispensable for achieving higher current densities necessary for fast charge of solid-state batteries. While this is often accomplished by soaking polymer electrolytes with enormous amounts of plasticizers, even exceeding the weight of dry membranes,^13−15,17,45^ the hybrid approach aims at reducing the addition or even completely avoiding plasticizers while simultaneously maintaining reasonably high ionic conductivity. Temperature-dependent ionic conductivities and solvent uptake (SU) of the hybrid membranes are displayed in Figure 3a,b. The SU was derived from the expression
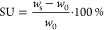
3where *w*_s_ and *w*_0_ denote the weight of the swollen and dry membranes, respectively. To better compare both the pristine polymer and hybrid electrolyte, a PSAb membrane was prepared for which the actual solvent uptake was limited to 51 wt %, comparable to hybrid electrolytes.

**Table 1:** Initial Ratios between PSAb and APTES-LATP (in wt %) Particles and Notation of the Resulting Membranes

notation	PSAb	PSAb5A	PSAb10A	PSAb20A	PSAb33A
PSAb:APTES-LATP	1:0	95:5	90:10	80:20	66.6:33.3

All of the hybrid membranes with APTES-LATP particles afford higher ionic conductivities compared to pristine PSAb at similar solvent uptake. For example, the composition PSAb20A with 20 wt % APTES-functionalized LATP particles yields 0.7 mS cm^–1^ at 40 °C, a value five times higher than in the case of PSAb reference. Nevertheless, the PSAb membrane with natural SU of 140% still offers the highest ionic conductivity above 1.4 mS cm^–1^, whereas the solvent uptake decreases gradually with higher amounts of APTES-LATP within the hybrid membranes. To better understand the benefits of adding APTES-LATP to polymer blends, hybrid membranes containing other inorganic particles such as Al_2_O_3_ and unmodified LATP were prepared (Figure 3c). As anticipated, even the inactive filler material Al_2_O_3_ enhances the ionic conductivity, most likely due to limited crystallinity of the polymer components, higher intermixing of polymer domains, or enhanced Li-salt dissociation due to Lewis acid–base interactions with the particles, thus resulting in an increased charge carrier concentration.^46^ In the cases of Al_2_O_3_ and unmodified LATP, no significant differences in overall ionic conductivity are observed, indicating that Li^+^ conduction through the bulk ceramic particles plays a negligible role. Instead, ion conduction at the interface between particles and polymer as well as that within the polymer matrix due to EC/PC solvation are the major contributions to the observable charge transport. Here, the surface modification of LATP with APTES and the associated change in ζ potential improves the interface *via* electrostatic attraction with the polymer PSA, yielding improved ionic conductivity. Note that similar effects were very recently reported for three-dimensional (3D) composite electrolytes comprised of APTES-LATP/PVdF, incorporated in PVC and LiTFSI.^47^

**Figure 4:**
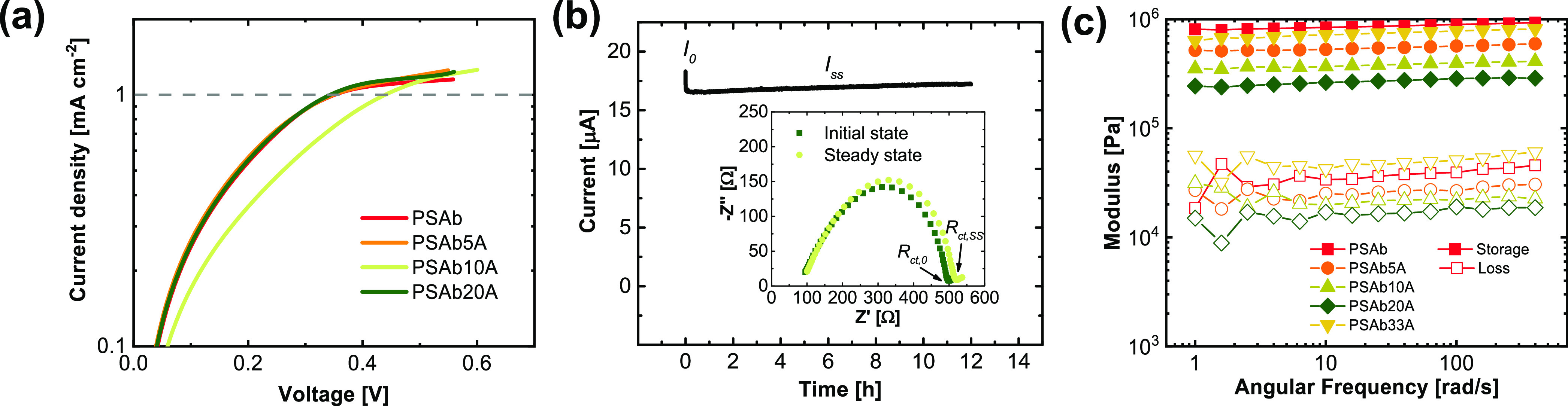
(a) Limiting current density measured *via* linear sweep voltammetry in Li||Li cells at 40 °C, (b) chronoamperometry and impedance spectroscopy measurement of the hybrid membrane PSAb20A, (c) storage (*G*′) and loss (*G*″) moduli of the hybrid membranes in comparison to pure polymer membrane at 40 °C; the membranes were placed with the oxide-rich side to the bottom on the substrate.

In addition, the limiting current density for all compositions was measured by linear sweep voltammetry, as shown in Figure 4a. Irrespective of actual LATP particle fractions, a limiting current density higher than 1 mA cm^–2^ was achieved at 40 °C, which is comparable to other “*quasi”*-solid electrolytes,^15,48^ corroborating that the Li reservoir of the LATP particles might be disconnected from the polymer domains. In theory, both materials containing Li^+^ are single-ion conductors and a value of 1 would be expected for *t*_Li^*+*^_. In practice, *t*_Li^*+*^_ varies between 0.85 and 0.95 due to the presence of remaining lithium base, water, or any other charge carriers (anions), which was also observed for other SIPE reported in the literature.^43,49^ Based on the technique proposed by Evans et al.,^43^ a value of 0.86 was determined for PSAb20A (Figure 4b), which is within the range of other reported SIPEs.

Besides ionic conductivity and Li^+^ transport, the mechanical properties of solid electrolytes are also very important. Therefore, rheology data was obtained for different membrane compositions to elucidate the impact of the particles within the membrane (Figure 4c). A frequency sweep was carried out at a constant strain amplitude of 0.05%. In all cases, the storage moduli (*G*′) are higher than the respective loss moduli (*G*″), displaying a solid-like behavior attributed to the high melting points of the polymer components. Upon addition of the particles to the membrane, both *G*′ and *G*″ are reduced to a minimum of 200 and 10 kPa for PSAbA20, respectively. In the case of PSAb33A, both values increase again and almost reach the value measured for pristine polymer membranes.

### 3.3 Detailed Characterization of PSAb20A Membrane Morphology

**Figure 5:**
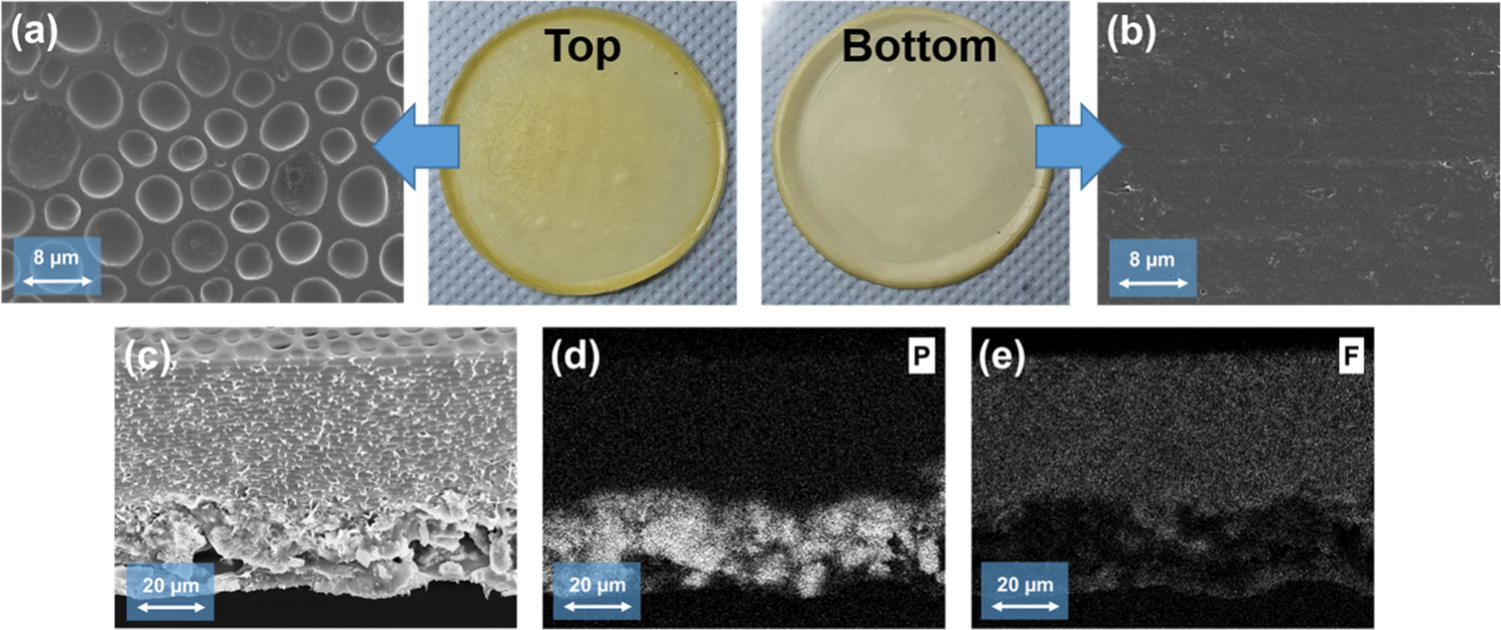
SEM images of the (a) top surface and the (b) bottom surfaces of the membrane together with images of the membrane; (c) cross section of the membrane and EDX mapping of (d) phosphorus and (e) fluorine from the membrane.

A detailed analysis of the membrane morphology and distribution of the particles was performed based on SEM measurements of the hybrid electrolyte PSAb20A, which exhibited the highest ionic conductivity. At the top side of the membrane (Figure 5a), pores of different sizes are recognizable, ranging from 2 to 8 μm, most likely due to the rigid aromatic structure of PSA, as was also observed in the literature.^50,51^ Moreover, no oxide particles are detected at the top surface of the membrane. In contrast, the bottom surface of the membrane (Figure 5b) appears whitish, indicating the presence of oxide particles. No micro- or nanopores are visible; instead, this part has a rather dense structure. Cross-sectional SEM images and EDX mapping (Figure 5c–e) revealed that the particles are not homogenously distributed within the membrane but rather concentrated at the bottom of the membrane. The increase of particle size after calcination due to aggregation as well as the slow-evaporating solvent NMP during membrane casting results in the formation of an oxide-rich layer with a thickness of 25–30 μm (total membrane thickness 90–110 μm) at the bottom of the Petri dish. The LATP particles are coated and connected with the polymer, but the mass fraction of polymer species (as monitored by fluorine mapping) is strongly reduced in this region compared to the residual parts of the membrane. It should be noted that the bottom layer is still covered by a thin polymer layer, as can be seen by an increased fluorine intensity at the bottom of Figure 5e. Note that the bottom layer was also studied *via* XPS, including etching for 240, 480, 720, and 960 s (etching depth of ∼3 nm for each sputtering step). Figure S9 shows the atomic concentration of the elements at the surface and after etching. The highest values are observed for C, O, and F, even after etching for 960 s, which are the main constituents of both polymers. The concentration of Al, Ti, and P increases only slightly after etching, and at some spots, an increase in intensity is observed, thus illustrating the presence of the polymer layer. This layer can enable sufficient contact with Li metal and protect LATP from Ti^4+^ reduction to Ti^3+^, which is otherwise often observed in the case of LATP in direct contact with Li metal.^52^

### 3.4 Electrochemical Analysis

**Figure 6:**
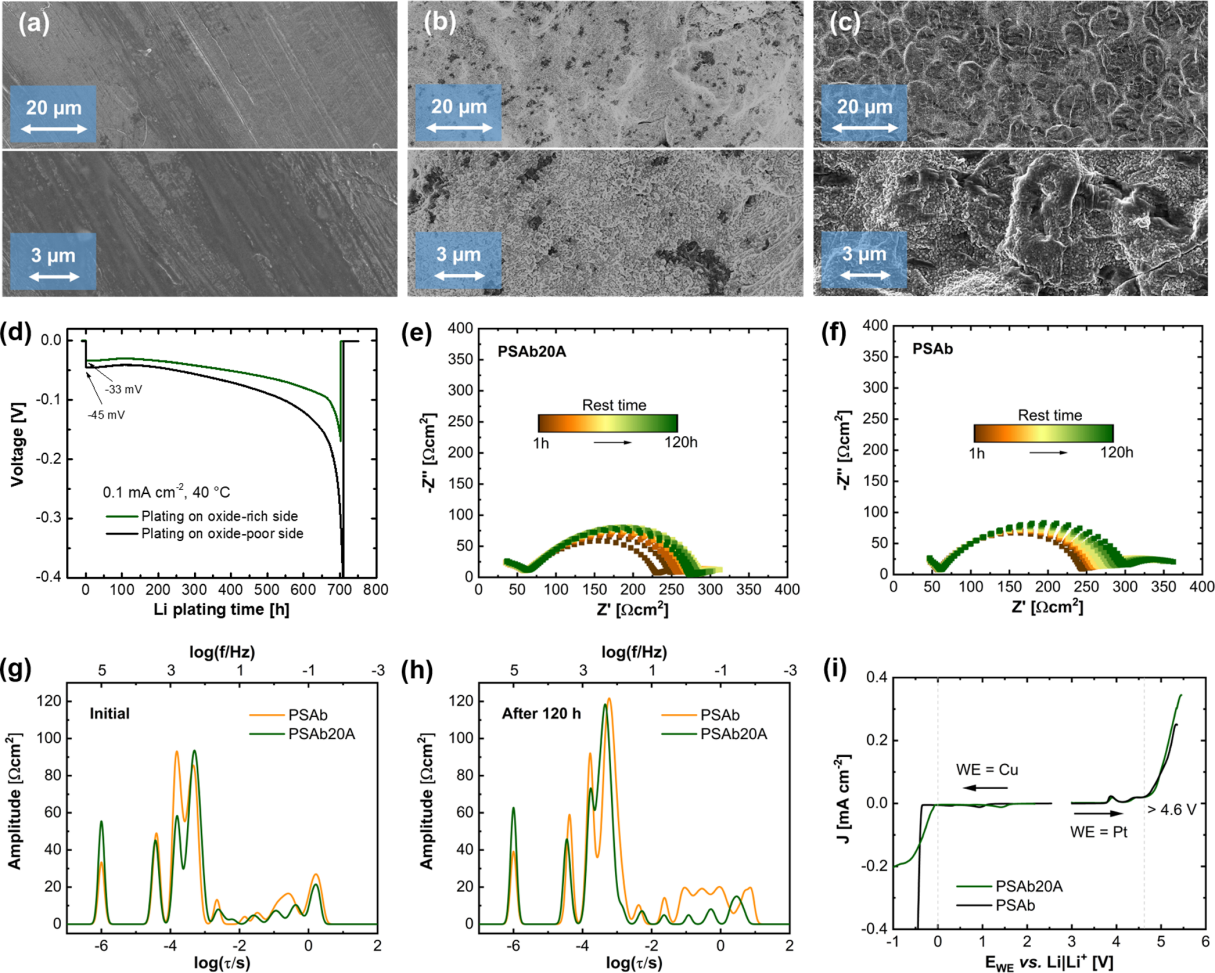
SEM images of (a) pristine Li metal, (b) Li metal facing the oxide-rich layer and (c) Li metal facing only polymer after 50 h of Li deposition at 0.1 mA cm^–2^; (d) comparison of single-side Li deposition in Li|PSAb20A|Li cells; EIS measurement of symmetric Li||Li cells over time at 40 °C with two sandwiched (e) PSAb20A membranes (oxide-rich side facing the Li metal) and (f) PSAb membranes; associated DRT spectra after (g) 0 h and (h) 120 h; (i) linear sweep voltammetry of PSAb20A and PSAb with Li metal as counter and reference electrodes and platinum and copper as the working electrode at room temperature. The impedances are normalized in units of Ω·cm^–2^ by a division of 2 accounting for the symmetric cell and multiplication of the electrode area.

Since the surface morphology of both sides of the introduced hybrid membrane differs, the behavior of those surfaces upon Li plating was deeply investigated. Single-side Li deposition was performed for 50 h at 0.1 mA cm^–2^ on a Li metal electrode facing the oxide-rich layer and an electrode facing the polymer layer. The results are compared to a fresh lithium metal electrode after roll-pressing in Figure 6. The lithium metal facing the oxide-rich side is covered with APTES-LATP as the uppermost layer of particles and the polymer is sticking to the Li metal surface when removing the hybrid membrane, masking almost the whole Li metal surface. Only close to the edges of the Li metal disk, several spots without particles on the electrode surface could be found (Figure 6b). While the roll-pressed Li metal surface is generally smooth and only has stripes from the roll-pressing process, both Li metal electrodes show some deposited Li in the form of small spheres in similar sizes after plating for 50 h. There is no obvious dendrite formation, probably due to the high ionic conductivity of the membranes and the single-ion conducting properties. However, the general morphology of those Li metal surfaces differs from each other. At the oxide-rich site, a more homogenous Li deposition is observed, only disturbed by some spots (dark color in the image) with slightly less deposited Li metal spheres. The Li metal surface facing the polymer is more inhomogenous, and it seems that Li deposition is favored at certain spots, resulting in a rougher surface morphology. In addition, Li deposition in Li|PSAb20A|Li cells was performed at a current density of 0.1 mA cm^–2^ until short-circuiting the cell. Figure 6d displays the voltage profile during Li deposition with the oxide-rich layer facing the plating side (green curve) and the stripping side (black curve) until short-circuiting of the cell occurs. The overpotential at the beginning of Li plating is −33 mV on the oxide-rich side, 27% smaller than that for Li deposition on the polymer-rich side (−45 mV). Pores at the top surface of the membrane probably result in decreased contact with Li metal and higher current densities at the remaining interfaces during Li plating, hence yielding higher overpotential. This observation is in agreement with the observed rougher surface (Figure 6c) structure of Li metal electrodes facing a porous polymer layer after Li deposition. The trend continues with further plating, and in both cases, an increase of the overpotential over time is noticed, either reflecting continuous SEI growth or roughening of Li metal surfaces upon deposition. Remarkably, Li plating is feasible for up to 700 h until a short circuit of the cell occurs, corresponding to 70 mAh cm^–2^ or 18.1 mg cm^–2^ of plated Li metal (when assuming a theoretical specific capacity of 3860 mAh g^–1^).^1^ Notably, in view of a determined mass of 21.1 mg cm^–2^ for the Li metal electrode, almost the whole electrode could be stripped during single-sided deposition. This explains the sudden increase in the overpotential prior to short circuit, highlighting a starting depletion of Li metal at the stripping side. Overall, the hybrid membrane can successfully withstand the volume changes of the electrodes, thereby mitigating the formation of Li “dendrites.” Additionally, XPS measurements of a lithium metal electrode facing the oxide-rich layer were compared with measurements of an electrode facing the polymer layer after 50 h of Li plating/stripping (Figure S10). On both surfaces, almost identical signals were detected, which can be attributed to the polymers PSA/PVDF-HFP, carbonate solvents, and LiF. While particles were observed on the Li metal surface facing the oxide-rich layer *via* SEM, no Al, Ti, or P signals were observed for XPS. Therefore, the particles and the Li metal surface are covered with the polymer or solvent, which hampers conclusions regarding potential LATP decomposition. As an alternative, the aging of the interface/interphase in contact with Li metal was monitored by EIS measurements (Figure 6e,f). Due to the different surfaces of the hybrid membrane, two PSAb20A membranes were sandwiched between Li metal so that the oxide-rich side contacts each electrode. A cell with two-stacked PSAb membranes was assembled for proper comparison. Both spectra display three (partially) completely depressed and deformed semicircles, indicating the presence of several processes with different frequency domains. The first semicircle reflects contributions from the bulk electrolyte (*R*_EL_) and is quite similar for both systems. A slight increase in the resistance can be observed over time (Figure S10), which is attributed to small changes in ionic conductivity, *e.g*., due to changes in solvent distribution. The second semicircle gradually increases for the reference system PSAb over time, but in the case of PSAb20A, it starts to decrease after 30 h, stabilizing at a value of ≈210 Ω·cm^–2^. To better understand the processes at the interface/interphase and to establish a suitable equivalent circuit model, distribution of relaxation time (DRT) analysis, which recently is becoming a more popular technique in battery research,^53−55^ was conducted initially and after 120 h resting time (Figure 6g,h). Here, the rates of individual processes are related to distinct time constants τ (τ ∝ 1/*f*, *f* is the frequency) and processes such as ion migration through the SEI or reactions at electrolyte|electrode interfaces can be reasonably distinguished. The DRT spectra are quite similar for both membranes, displaying three authentic peaks in the domain between τ = 10^–6^–10^–3^ s. With the first peak having a time constant of τ = 10^–6^ s, the underlying process can be attributed to migration in the bulk electrolyte. The second one can be associated with ion migration through interphases, *e.g.*, the SEI layer, while the last one most likely reflects charge transfer processes.^54,56,57^ Interestingly, after 120 h of resting time, a small shift of these two DRT peaks to lower time constants is observed for PSAb20A compared to PSAb (indicated by the arrows), illustrating slightly improved or faster processes for PSAb20A membranes. Moreover, the second peak remains almost unchanged over the aging period for PSAb20A, which signalizes a robust interphase without ongoing side reactions. Based on the DRT analysis, an equivalent circuit model with four R-CPE elements was fitted for the EIS data, which is shown in Figure S12. Indeed, the overall cell resistance for PSAb20A is slightly smaller compared to PSAb at the beginning, and the difference between both cells is increasing over the aging period due to the stabilization of the second semicircle in the case of PSAb20A.

In practice, for cycling of the hybrid membranes in NMC622||Li full cells, high oxidative stability is necessary. Therefore, linear sweep voltammetry was carried out to derive the hybrid electrolyte’s electrochemical stability window (ESW) (Figure 6i). The reductive scan shows metallic Li plating at potentials <0 V *vs* Li|Li^+^ and minor peaks at 1.0–1.5 V *vs* Li|Li^+^, reflecting minor electrolyte decomposition of EC/PC.^58^ During the oxidative scan, a peak is present at 3.9 V *vs* Li|Li^+^ in both systems, most likely indicating residual impurities stemming from polymer synthesis, *e.g*., lithium base LiHMDS. This is further supported by NMR measurements of the lithiated polymer, where an additionally observed peak at 0 ppm highlights the presence of the base (Figure S6). Other than that, exponential oxidation of the membranes is observed at potentials > 4.6 V *vs* Li|Li^+^, rendering them attractive materials for application with high-voltage cathode materials, such as LiNi_0.*x*_Mn_0.*y*_Co_0.*z*_O_2_ or LiNi_0.*x*_Co_0.*y*_Al_0.*z*_O_2_. However, it should be noted that inert electrodes employed for the determination of the ESW do not necessarily display the actual ESW of the materials in full-cell configuration and under operation with active materials.^59^

**Figure 7:**
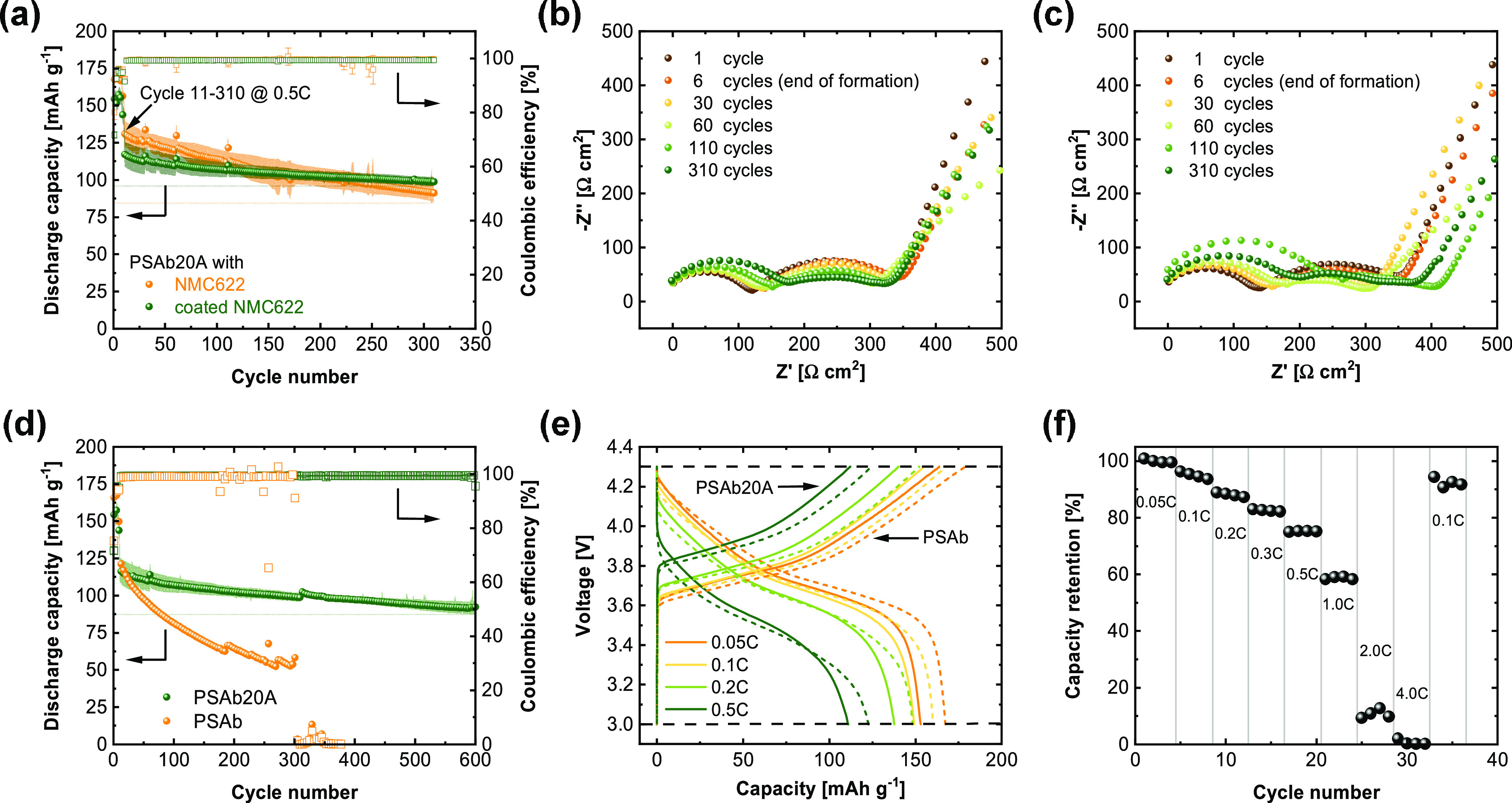
(a) Long-term cycling performance of NMC622|PSAb20A|Li cells with coated and noncoated NMC622 and their corresponding Nyquist plots ((b) coated and (c) noncoated NMC622) measured *via* EIS at the end of selected cycles; (d) long-term cycling performance of PSAb20A hybrid electrolytes in comparison to PSAb in coated NMC622||Li full cells and (e) their voltage curves of selected cycles; (f) capacity retention of PSAb20A in coated NMC622||Li cells. All experiments were done at a temperature of 40 °C, with the following cycling procedure for (a, d) 2 × 0.05C, 2 × 0.1C, 2 × 0.05C, 2 × 0.1C, 2 × 0.2C followed by cycling at 0.5C (≙ 0.18 mA cm^–2^).

Therefore, the performance of the hybrid electrolyte was further elucidated based on long-term constant current cycling and rate capability measurements in NMC622||Li full cells. In view of its high glass-transition temperature, an annealing step after cell assembly, commonly done for polymer systems such as PEO, is ineffective. To enable sufficient contact with the cathode active material, the cathode was spin-coated with 30 μL of a solution containing 10 wt % PSA in DMAc and dried afterward as reported previously.^60^ By application of oxidic coatings, in particular, LiNbO_3_, onto the NMC active material, the achievable capacity retention of battery cells with solid or liquid electrolytes may be significantly improved.^61−63^ To investigate the influence of such cathode coatings onto the cell performance with composite electrolytes, commercial NMC622 as well as LiNbO_3_-coated NMC622 (c-NMC622) was subjected to constant current cycling at 0.5C and 40 °C (Figure 7a). The achievable discharge capacity of as-obtained NMC622 in the first cycles and at the beginning of the long-term cycling amounts to 131 mAh g^–1^ at 0.5C, which is higher than that in the case of cells that are operated with c-NMC622 (118 mAh g^–1^). However, the overall capacity retention of cells containing c-NMC622 is superior, as revealed by merely 16% capacity losses after 300 cycles. At the end of several cycles, EIS data was recorded for cells with c-NMC622 (Figure 7b) and as-obtained NMC622 (Figure 7c), the fitting, as well as an equivalent circuit, is shown exemplary after the first cycle in Figure S13a,b. In both cases, a constant increase of the first semicircle (R1) over cycle numbers is observed (Figure S13c), which can be attributed to ion migration through interfaces, such as SEI/cathode electrolyte interphase (CEI^64^) due to the high frequency of 100 kHz. The increase of R1 is most likely caused by ongoing decomposition and a growing SEI/CEI. However, R1 and the further increase of the resistance during cycling are lower for the cell with c-NMC622 is slightly lower compared to commercial NMC622, which might be due to less decomposition of the solid electrolyte. The second semicircle is attributed to charge transfer processes at the electrode interfaces and is even decreasing for cells cycled with c-NMC622, reflecting an improvement of the interface toward the cathode active material upon cycling. At low frequencies, the typical Li^+^ diffusion in the cathode material is observed for both cells. Overall, better cycling performance is achieved when utilizing thin LiNbO_3_ coatings, which initially reduces the discharge capacities in the presence of additional layers that Li^+^ ions have to migrate through, but upon cycling form robust interfaces, thereby affording higher capacity retention in the long run. To better understand the impact of the hybrid electrolyte on the observable cycling performance of c-NMC622||Li cells, a cell containing a PSAb membrane, which was soaked in an excess of EC/PC for 48 h, was assembled for comparison (Figure 7d). The cells with the PSAb20A electrolyte exhibit much better capacity retention and can be cycled over 600 cycles without short-circuiting the cell, while the cell with PSAb suffers from severe capacity fading that results in cell failure at around 300 cycles. Besides the influence of the oxide-rich layer at the Li metal side, the “softer” polymer phase at the cathode might positively influence capacity retention by preventing active material losses upon cycling. Also, the choice of oxide particles within the hybrid system has a significant impact on the electrochemical performance of the respective cells. As shown in Figure S14, implementation of Al_2_O_3_ or unmodified LATP has no beneficial or even a worsening effect compared to the PSAb membrane. It should be noted that all of the cells with PSAb20A show a slight increase of the specific capacities after 310 cycles, arising from the extension of the cycling procedure and introduction of a small rest step that reduces the overall polarization effects in the cells. The voltage profiles (Figure 7e) at current densities of 0.05C, 0.1C, 0.2C, and 0.5C are displayed for cycles 6, 8, 10, and 12, respectively. Therefore, higher discharge capacities for cells operated with PSAb membranes are observed, which most likely represents the much higher solvent uptake of pristine polymer membranes and, hence, better wetting of the cathode active materials. Nevertheless, solely the PSAb20A membrane can establish a stable long-term cycling performance, preventing short circuits in the cells. In addition, upon inspection of the evolution of the voltage hysteresis during cycling (Figure S15), a continuous increase paired with strong capacity fade can be observed for the cells containing the PSAb membrane. In contrast, the cells with the PSAb20A membrane display almost no change in voltage hysteresis during cycling, indicating a robust interphase, less side reactions, and only a minor increase of the cell resistances. Figure 7f displays the capacity retention of c-NMC622|PSAb20A|Li full cells. At increased current flows, the achievable capacity retention decreases gradually, whereas at C-rates higher than 0.5C, a more severe drop in capacity retention is observed. While the measured ionic conductivity of 0.7 mS cm^–1^ at 40 °C and a limiting current density > 1 mA cm^–2^ should allow proper cycling at higher C-rates, recent findings indicate that in addition to contact issues to active materials, cell polarization effects are present even in the case of single-ion conductors so that slow Li^+^ diffusion through the SEI layers eventually becomes a bottleneck of cell operation at higher current densities.^65^ Here, indeed, an optimization of interfaces by introducing SEI-forming electrolyte additives and/or modification of electrode compositions, *e.g*., by the addition of more mobile and flexible oligomers into the composite cathode, might increase the available specific capacities even at higher current densities or enable higher mass loadings.^66−69^

## 4 Conclusions

In this work, an approach for designing hybrid electrolytes based on single-ion conducting polymers and surface-functionalized LATP particles by solution-casting is introduced and evaluated. Upon slow solvent evaporation, ceramic particles accumulate at the bottom of the hybrid membrane, yielding a robust oxide-rich layer. This concept provides a straightforward strategy to create layered systems that enable good contact among the major phases of the hybrid electrolyte. Though the presence of unmodified LATP or Al_2_O_3_ to PSAb somewhat promotes the ionic conductivity, the addition of APTES-functionalized LATP particles yields a significant enhancement, which is attributed to electrostatic interactions with the polymer. Despite that a fraction of EC/PC is required to boost the achievable Li^+^ ion transport, the mobilizer uptake could be distinctly decreased to merely 38 wt % in the case of hybrid electrolytes containing 20 wt % APTES-LATP while maintaining a reasonable ionic conductivity of 0.7 mS cm^–1^ at 40 °C. Furthermore, the introduced hybrid electrolyte allows for single-sided Li deposition for over 700 h at a current density of 0.1 mA cm^–2^ in Li||Li symmetric cells, corresponding to 18.1 mg cm^–2^ of plated Li, without short-circuiting the cells due to “dendrite” penetration or deformation of the polymer/oxide hybrid membranes, by far exceeding Li plating time of cross-linked PEO (>16 h at 0.2 mA cm^–2^).^70^ For potential industrial applications, compatibility of the hybrid electrolytes with high-voltage cathode materials at moderate and higher C-rates is essential. Many current systems reported in the literature exploited polymers such as PEO or PVdF-HFP together with oxide materials such as LATP or LLZO, though merely against cathode materials such as LiFePO_4_ or at significantly lower current densities/cycle numbers for NMC cathodes. Here, a polymer/oxide hybrid electrolyte is operated in NMC622||Li full cells at C-rates of 0.5C, thereby yielding robust capacity retention upon cycling. When exploiting NMC622 particles with a thin LiNbO_3_ coating as a protective layer, the cycling stability is even more enhanced to an overall capacity retention of 78% after 600 cycles. However, the polymer’s reduced solvent uptake and high glass-transition temperature eventually limit the contacts to the cathode active material, even after enhancing contacts by spin-coating the cathode with a polymer-containing solution. At higher current densities, interfacial resistances yield overpotentials that restrict the accessible specific capacity from the composite cathodes. Further improvement of the charge transfer kinetics, *i.e.*, by adding mobile oligomers to the cathode, is expected to increase the achievable specific capacities and/or even afford higher cathode mass loadings.

## Supporting Information Available

Polymer synthesis; exemplary NMR spectra; GPC of polymer; TEM image and size distribution of LATP before annealing; XPS data of the PSAb20A membrane and Li metal after cycling; EIS data of Li|Li and (c-)NMC622 cells with PSAb and PSAb20A; and cycling performance of PSAb with Al_2_O_3_ and LATP (PDF)
