# Supplementary material for: Ceramic-in-Polymer Hybrid Electrolytes with Enhanced Electrochemical Performance
Source: ACS Appl Mater Interfaces. 2022 Nov 21;14(48):53636–47. doi: 10.1021/acsami.2c13408 (PMC9743088; doi:10.1021/acsami.2c13408)
Supplement: Supplementary file 1 — am2c13408_si_001.pdf [file am2c13408_si_001.pdf]

Supporting information

Ceramic-in-polymer hybrid electrolytes with enhanced electrochemical performance

Gerrit Michael Overhoff [1], Md Yusuf Ali [2], Jan-Paul Brinkmann [1], Peter Lennartz [1], Hans Orthner [2], Mohaned Hammad [3], Hartmut Wiggers [2,4], Martin Winter [1,5], Gunther Brunklaus [1,]*

1 Helmholtz Institute Münster, IEK-12, Forschungszentrum Jülich GmbH, Corrensstr. 46, 48149 Münster, Germany

2 Institute for Combustion and Gas Dynamics—Reactive Fluids, University of Duisburg-Essen, Carl-Benz-Straße 199, 47057, Duisburg, Germany

3 Institute for Combustion and Gas Dynamics—Particle Science and Technology, University of Duisburg-Essen, Carl-Benz-Straße 199, 47057, Duisburg, Germany

4 CENIDE, Center for Nanointegration, University of Duisburg-Essen, Carl-Benz-Straße 199, 47057, Duisburg, Germany

5 University of Münster, MEET Battery Research Center, Institute of Physical Chemistry, Corrensstr. 46, 48149 Münster, Germany

<u>*Corresponding Author:</u> Gunther Brunklaus (g.brunklaus@fz-juelich.de)

Synthesis of polymer PSA

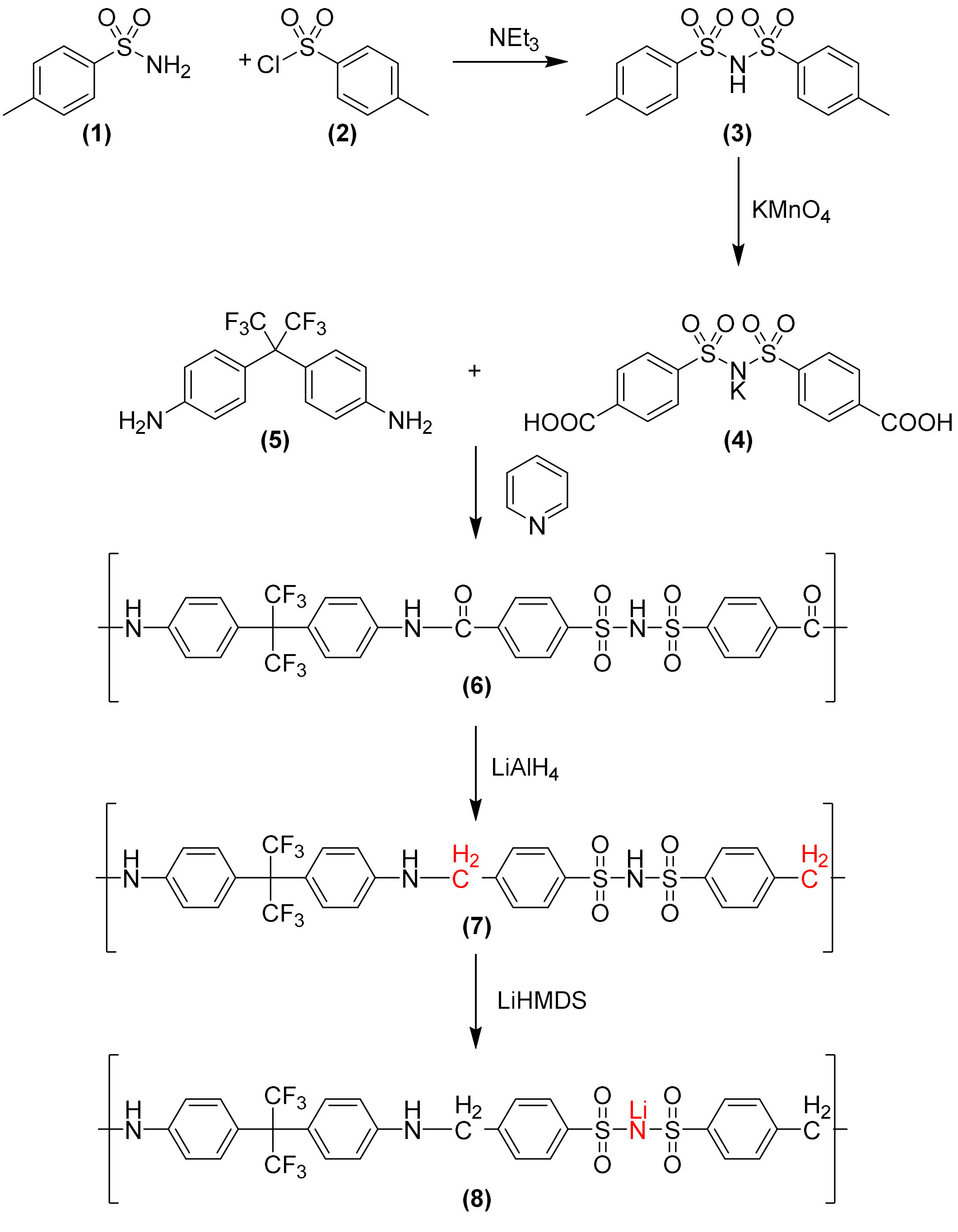

Figure S1: *Synthesis route for the single-ion conducting polymer PSA* ***(8)***.

Synthesis of bis(4-methyl benzene sulphonyl)imide (3)

P-toluenesulfonyl chloride **(2)** (55.702 g, 292.03 mmol, 1 eq.) and p-toluenesulfonyl amide **(1)** (50.003 g, 292.03 mmol, 1 eq.) were dissolved in 150 mL anhydrous acetonitrile under argon atmosphere. Subsequently, triethylamine (82 mL, 591.56 mmol, 2 eq.) was slowly added to the reaction mixture, and the solution was stirred at 75 °C for two days. Then, the dark brown mixture was allowed to cool down to room temperature, and $HNEt_3Cl$ was filtered off. The solvent was removed *via* rotary evaporation,

and some deionized water was added to the dark brown oil. The solution was poured into HCl (2 M), resulting in a pale brown solid precipitation. The precipitate was collected and washed with 2 L of water over a filter. Accordingly, the desired product dissolved while brown impurities remained on the filter. The product was again precipitated using HCl, filtered, and washed twice with DCM and once with a small amount of water. The final product was obtained as a white powder with 71% yield (67.5 g).
1H NMR (400 MHz, DMSO-d6) δ [ppm]: 7.52 (d, 4H, Ar-H), 7.16 (d, 4H, Ar-H), 2.32 (s, 6H).

Synthesis of potassium(4-carboxyl benzene sulphonyl)imide monomer (4)

Bis(4-methyl benzene sulphonyl)imide **(3)** (15.002 g, 46.10 mmol, 1 eq.) was added to 500 mL of deionized water. The suspension was heated to 95 °C, and lithium hydroxide monohydrate (1.938 g, 46.10 mmol, 1 eq.) was added. Potassium permanganate (36.423 g, 230.49 mmol, 5 eq.) was slowly added to the solution, and the reaction was stirred overnight under reflux. The reaction was stopped, manganese dioxide was filtered off, and the filter was washed with 1 L of water. Hydrochloric acid was added to the clear filtrate until the solution reached a pH-value of 1, leading to a white solid's precipitation. Finally, the collected solid was washed with 1 L methanol and dried under reduced pressure (10^{-3} mbar) at 80 °C to obtain the product as a white powder with a yield of 84% (16.4 g).
1H NMR (400 MHz, DMSO-d6) δ [ppm]: 13.16 (s, 2H), 7.91 (d, 4H, Ar-H), 7.73 (d, 4H, Ar-H).

Polycondensation: Synthesis of polymer (6)

First, calcium chloride (4.210 g, 38.02 mmol, 3.2 eq.), triphenylphosphite (19.571 g, 63.05 mmol, 5.3 eq,), potassium(4-carboxyl benzene sulphonyl)imide **(4)** (5.009 g, 11.81 mmol, 1 eq.) and 4,4-(hexafluoroisopropylidene)dianiline **(5)** (3.950 g, 11.81 mmol, 1 eq.) were dissolved in 40 mL dry N-methyl-2-pyrrolidone. Then, pyridine (8.50 mL, 105.42 mmol, 9 eq.) was added to the mixture, and the reaction mixture was heated up to 110 °C for 8 h. Subsequently, the reaction was stopped, and the solution was allowed to cool down to room temperature. Another 40 mL of N-methyl-2-pyrrolidone was then added to the viscous mixture. Afterwards, the solution was poured dropwise into 600 mL of methanol (placed in an ice bath). The white precipitate was filtered off, and the white sticky solid was washed several times with distilled H_2O to remove inorganic salts until a white powder was received. The substitution of potassium with hydrogen ions was performed by dissolving the polymer (7.60 g, 10.74 mmol) in 200 mL dimethyl sulfoxide. Afterwards, 2M hydrochloric acid was added dropwise until a white solid precipitates from the solution. The precipitate was filtered, and the process was repeated one more time. The final product was obtained as a white powder after washing with water and drying under reduced pressure (10^{-3} mbar) at 80 °C with a yield of 91%.
1H NMR (400 MHz, DMSO-d6) δ [ppm]:10.60 (s, 2H, C(=O)NH), 8.04-7.76 (m, 12H, Ar-H), 7.36 (d, 4H, Ar-H).

Reduction of the polymer: Synthesis of polymer (7)

Polymer **(6)** (2.000 g, 2.92 mmol) was dissolved in 100 mL anhydrous THF at room temperature. Subsequently, $LiAlH_4$ (0.520 g, 13.92 mmol) was slowly added, the solution heated up to 80 °C, and the reaction continued under reflux for 72 h yielding a greyish/greenish suspension. The reaction was stopped by adding some water into the suspension to react with residual $LiAlH_4$, and 2M HCl was added to the solution resulting in the precipitation of a white solid. The precipitate was filtered off, washed with water, and dried under reduced pressure (10^{-3} mbar) at 100 °C to yield the product as a fine powder (75%, 1.44 g).
1H NMR (400 MHz, DMSO-d6) δ [ppm]: 7.62 (d, 4H, Ar-H), 7.35 (d, 4H, Ar-H), 7.00 (d, 4H, Ar-H), 6.61 (d, 4H, Ar-H), 4.28 (s, 4H, CH_2).

Lithiation of the polymer: Synthesis of polymer (8)

Polymer **(7)** (1.10 g, 1.64 mmol, 1 eq.) was dissolved in 150 mL anhydrous tetrahydrofuran under an argon atmosphere. A 1M lithium bis(trimethylsilyl)amide solution in tetrahydrofuran (1.46 g, 1.64 mmol, 1 eq.) was added dropwise, resulting in precipitation of the lithiated polymer. The reaction mixture was left to stir overnight. The lithiated polymer was collected by filtration, dried under reduced pressure (10^{-3} mbar) overnight, and dried under high vacuum (10^{-6} mbar) at 80 °C for 5 days. The lithiation degree was controlled *via* ICP-OES measurement (ratio between lithium and sulfur).

1H NMR (400 MHz, DMSO-d6) δ [ppm]: 7.62 (d, 4H, Ar-H), 7.33 (d, 4H, Ar-H), 7.00 (d, 4H, Ar-H), 6.59 (d, 4H, Ar-H), 4.27 (s, 4H, CH_2).

NMR spectra of synthesized materials

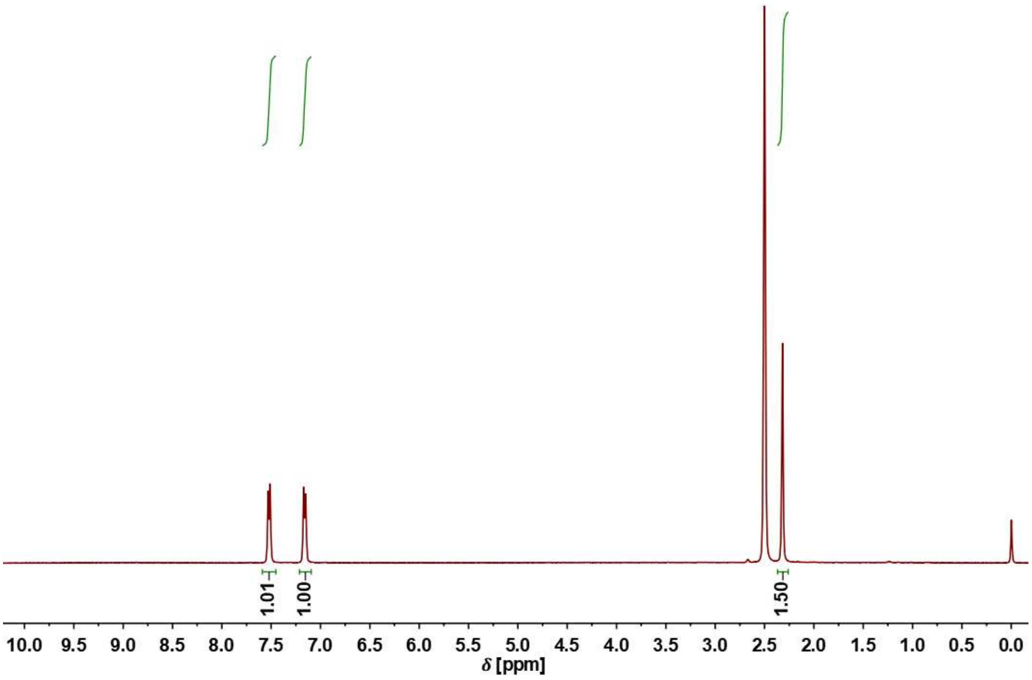

Figure S2: *Exemplary* ^{1}*H NMR spectrum of bis(4-methyl benzene sulphonyl)imide* ***(3)****, as measured in DMSO-d6.*

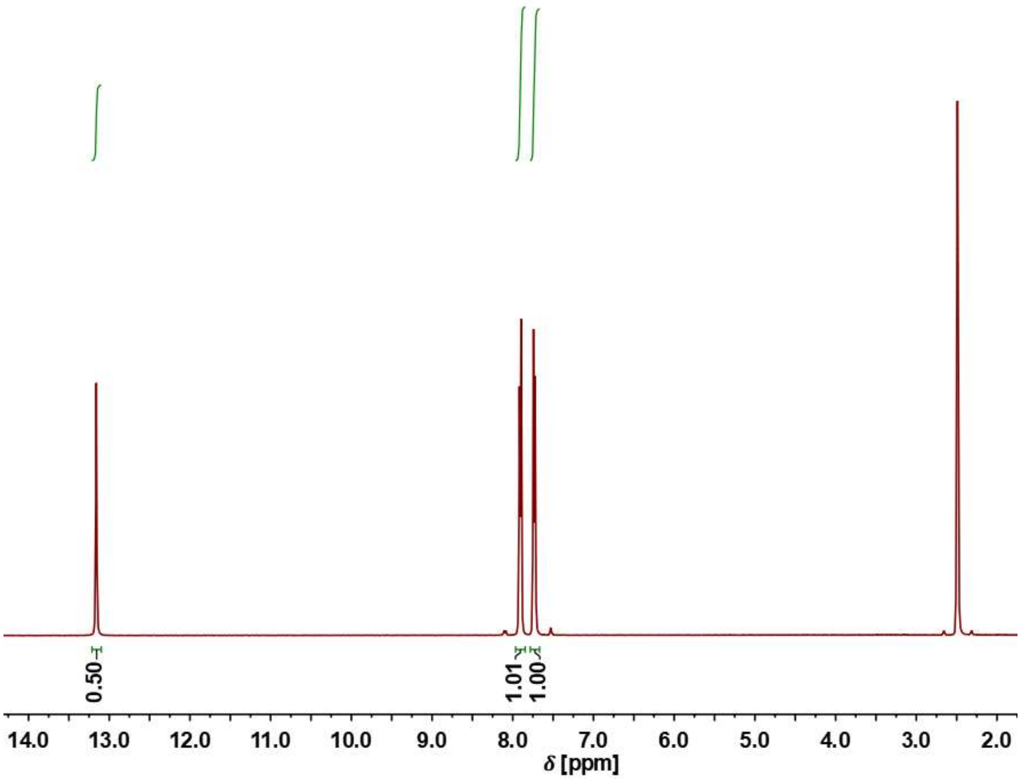

Figure S3: *Exemplary* [1]*H NMR spectrum of potassium(4-carboxyl benzene sulphonyl)imide monomer* ***(4)****, as measured in DMSO-d6.*

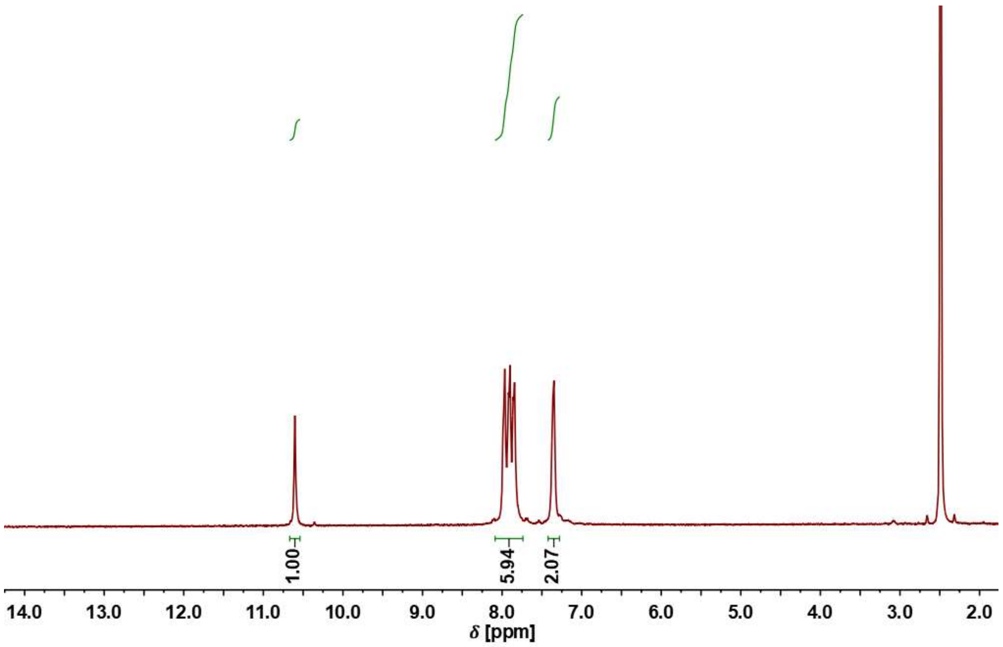

Figure S4: *Exemplary* [1]*H NMR spectrum of polymer* ***(6)****, as measured in DMSO-d6.*

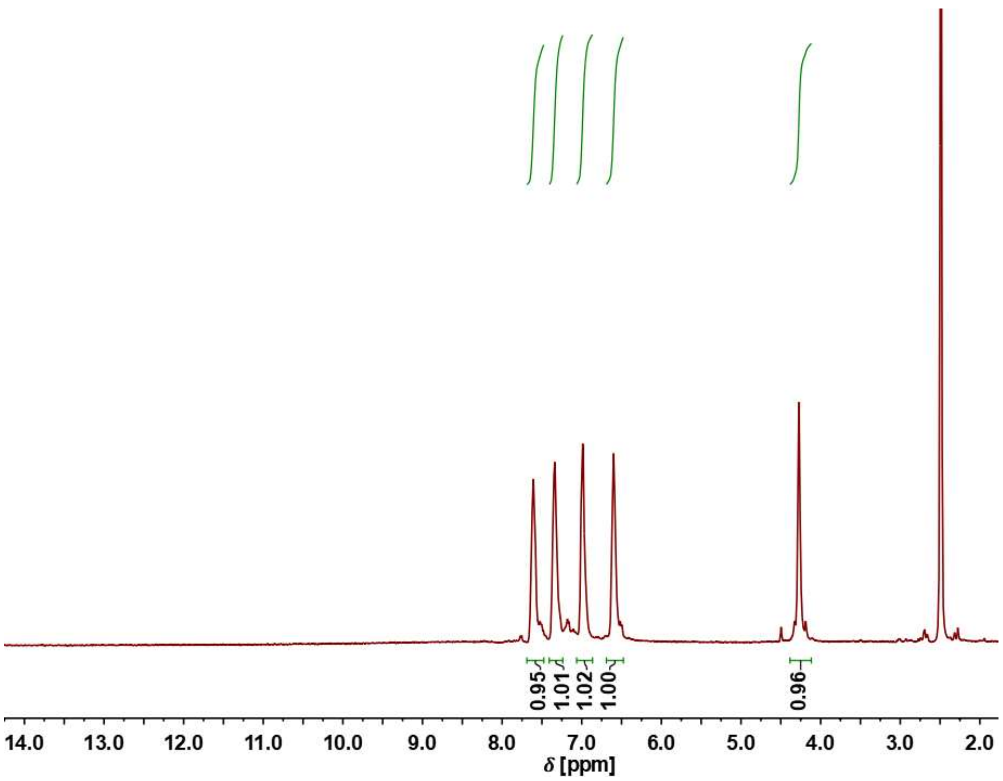

Figure S5: *Exemplary* ^{1}*H NMR spectrum of polymer* ***(7)****, as measured in DMSO-d6.*

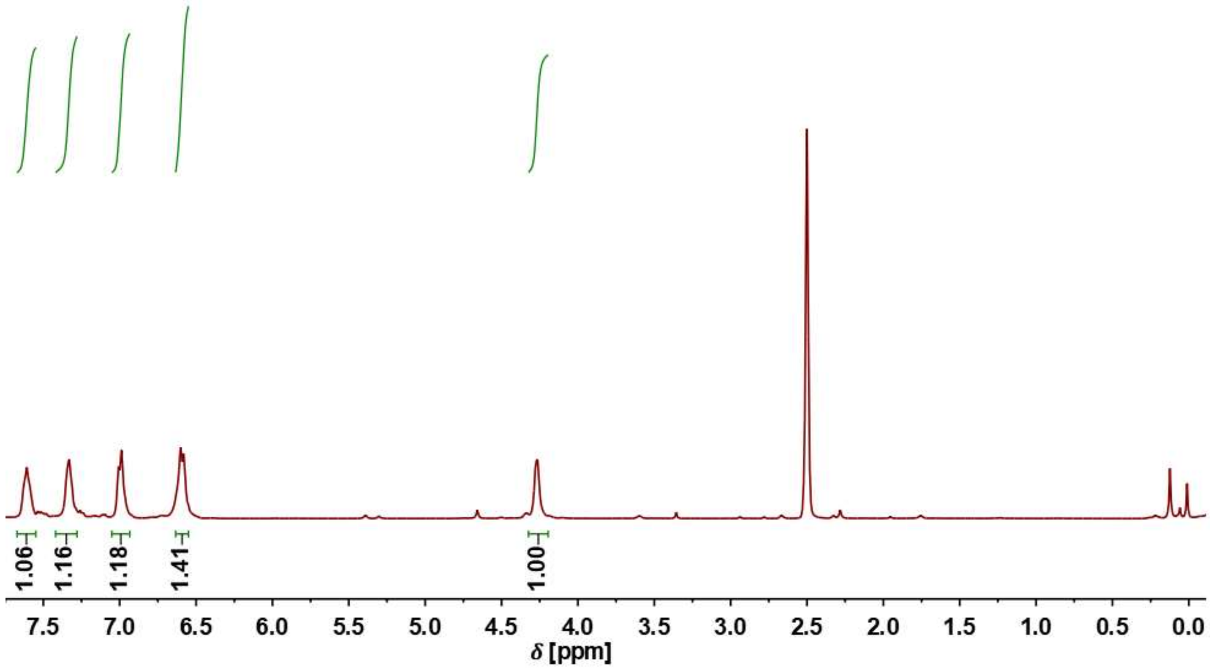

Figure S6: *Exemplary* ^{1}*H NMR spectrum of polymer PSA* ***(8)****, as measured in DMSO-d6.*

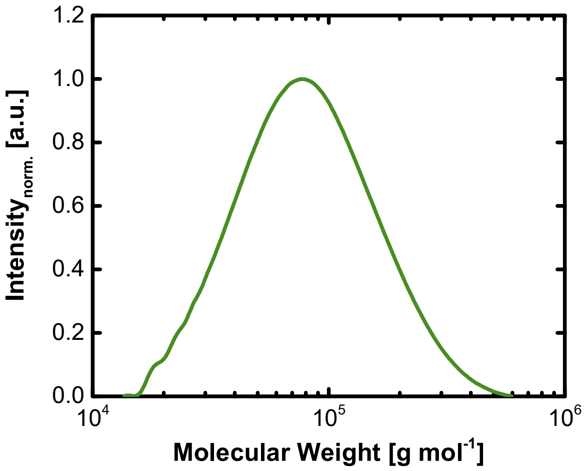

Figure S7: *GPC/SEC chromatogram showing the molecular weight distribution of polymer PSA* ***(8)*** *in DMAc (M_n = 64.950 g mol^{-1}, M_w = 98.490 g mol^{-1}).*

For measuring the molecular weight of the polymer **(8)**, an Agilent 1260 Infinity (*Agilent Technologies*) instrument equipped with PolyPore columns and a differential refractive index (RI) detector was used. N,N-dimethylacetamide containing 0.03 wt.% LiBr was utilized at 50 °C with a flow rate of 1 mL min^{-1} and the SEC system was calibrated using linear poly(methyl methacrylate) standards ranging from 800 to 2.2x10^6 g mol^{-1}. Typically, 100 μL of a 1.0 mg mL^{-1} sample solution was injected onto the columns.

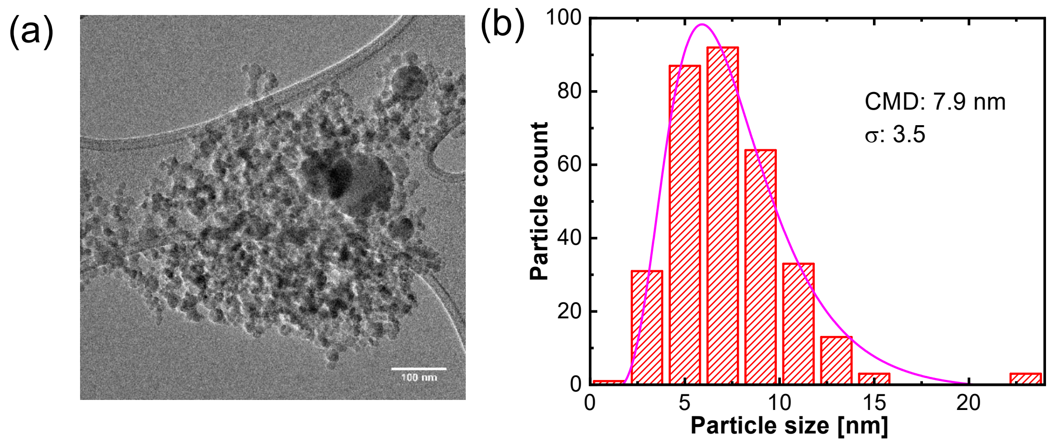

Figure S8*: Size distribution of LATP particles measured after SFS (but before calcination) by counting particles in TEM images.*

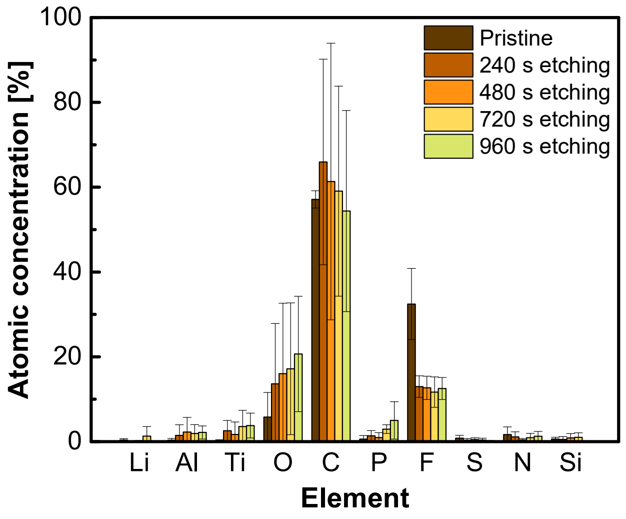

Figure S9: *Atomic concentration of different elements at the surface of the bottom membrane and after etching.*

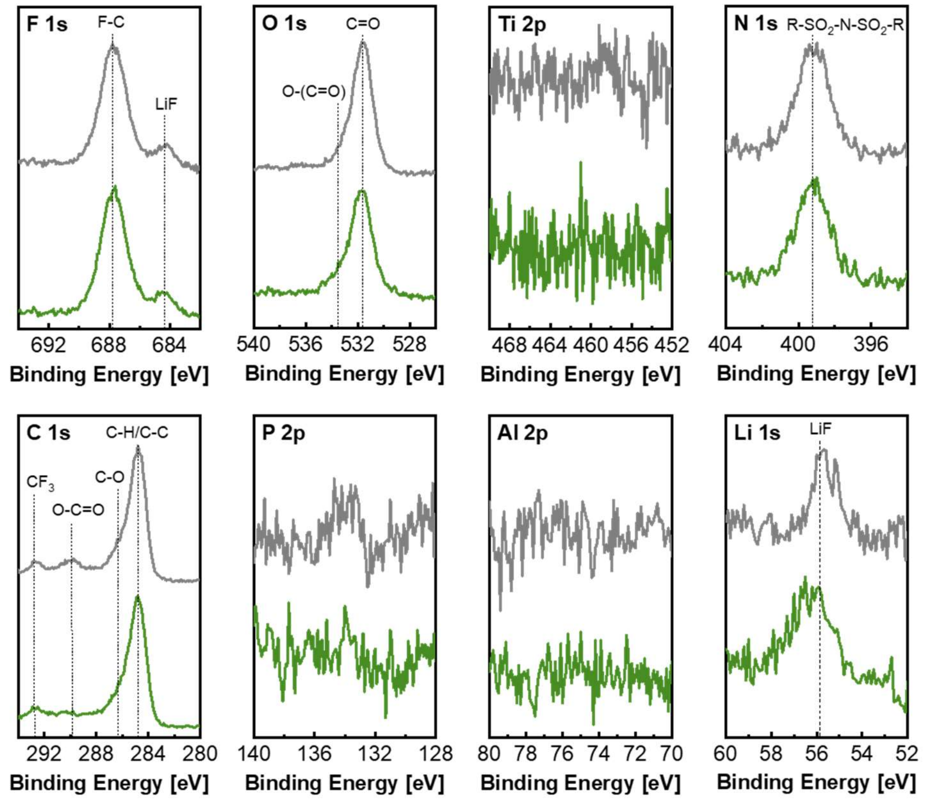

Figure S10: *XPS measurements of Li metal electrodes facing the oxide-rich (green) or the polymer-rich layer (grey) of a Li|PSAb20A|Li cell after 50 hours of Li plating/stripping at a current density of 0.1 mA cm*$^{-2}$ *for 1 hour per cycle. The electrodes were not washed before the measurements.*

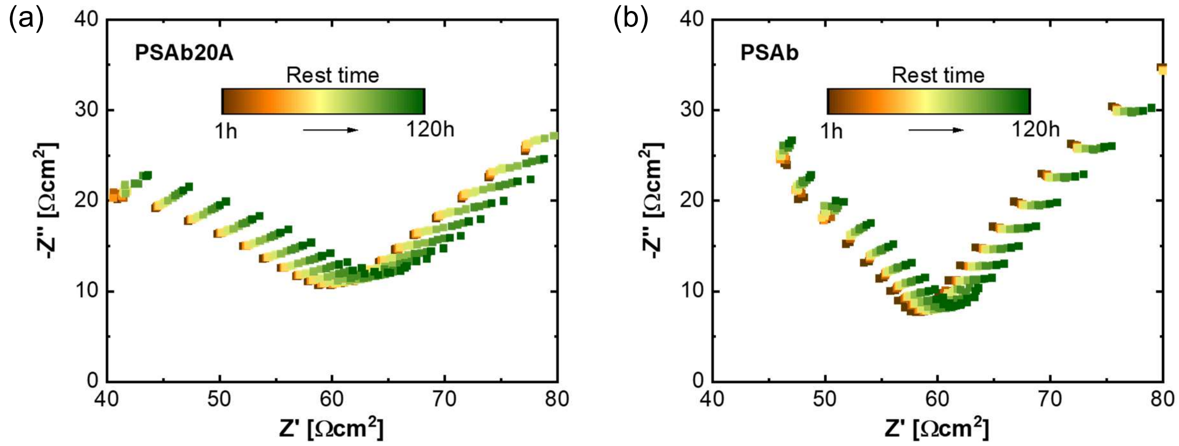

Figure S11: *EIS measurement of symmetrical Li|Li cells overtime at 40 °C with two sandwiched a) PSAb membranes and b) PSAb20A membranes (oxide-rich side facing the Li metal). The graphs show an increase of* R_{EL} *+* R_{SEI} *for both systems over time. The impedances are normalized in units of Ω* cm^{-2} *by division of 2 for accounting for the symmetrical cell and multiplication of the electrode area.*

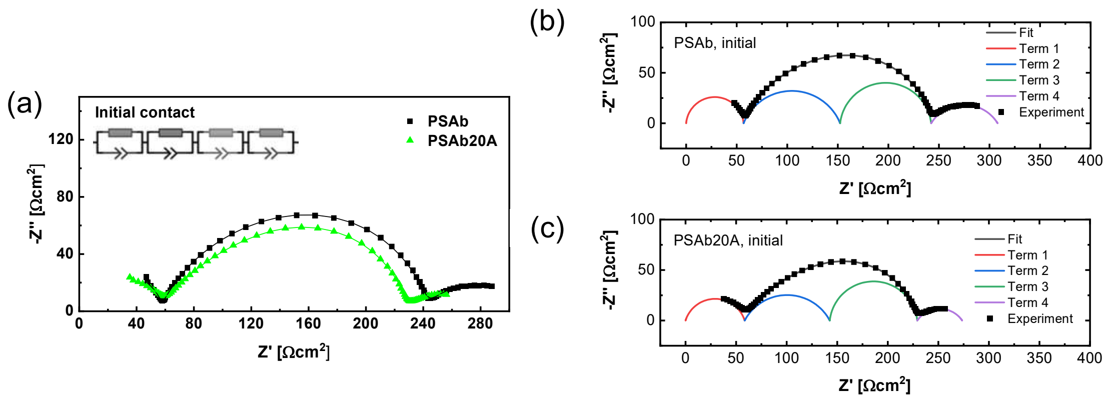

Figure S12: *Initial EIS measurement of symmetrical Li|Li cells overtime at 40 °C of PSAb and PSAb20A and their fitted spectra using 4 R-CPE elements. The impedances are normalized in units of Ω* cm^{-2} *by a division of 2 for accounting for the symmetrical cell and multiplication of the electrode area.*

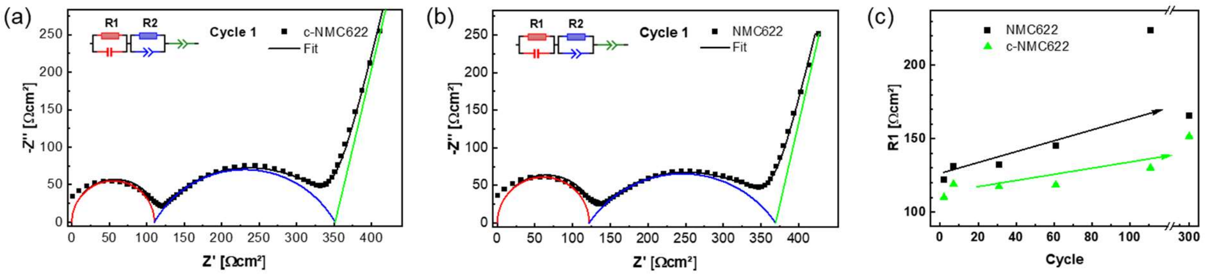

Figure S13: *Fitted EIS spectra and equivalent circuit models of NMC622|PSAb20A|Li cells with (a) LiNbO$_3$-coated NMC622 and commercial NMC622 cathode active material; (c) growth of R1 (red circle) with the cycling number.*

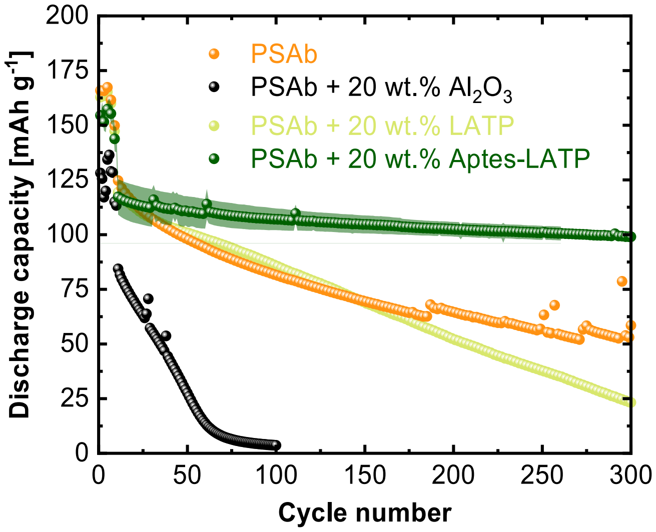

Figure S14: *Cycling performance of different hybrid membranes in coated NMC622|Li cells at 0.5C and 40 °C. It is demonstrated that only the hybrid membrane with APTES-LATP enables sufficient capacity retention during long-term constant current cycling.*

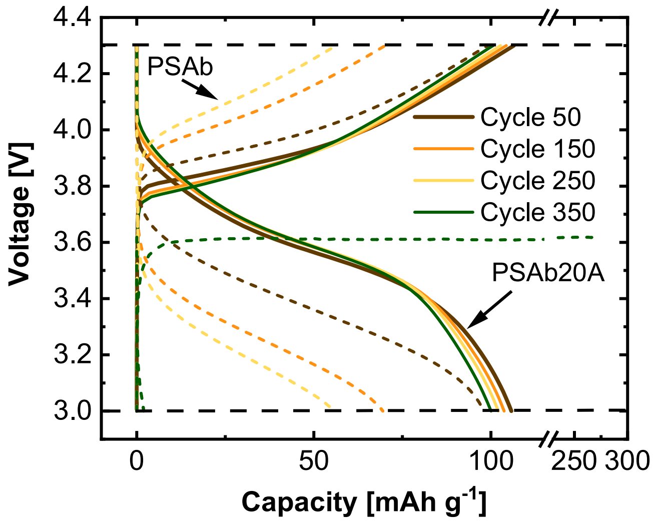

Figure S15: *Voltage curves of selected cycles of PSAb and PSAb20A in cells with coated NMC622 and Li metal at 0.5C and 40 °C. The cells operated with PSAb20A display a very stable cycling performance, loosing almost no capacity over the range of 300 cycles and no increase of the voltage hysteresis. In contrats, PSAb cells suffer under continous capacity fading and increase of the voltage hysteresis, resulting in complete cell failure (no discharge capacity in cycle 350).*
